# Supplementary material for: Experiences of trial participants and site staff of participating in and running a large randomised trial within fertility (the endometrial scratch trial): a qualitative interview study
Source: BMJ Open. 2021 Sep 16;11(9):e051698. doi: 10.1136/bmjopen-2021-051698 (PMC8449983; doi:10.1136/bmjopen-2021-051698)
Supplement: Supplementary data [file bmjopen-2021-051698supp002.pdf]

Endometrial Scratch Trial Staff Topic Guide

NOTES: This topic guide is a flexible tool and may be revised as new areas of interest arise during the process of data collection. The wording of questions is for guidance only and can be varied to suit the natural style of the interviewer and the level of understanding of the participant.

ITEMS REQUIRED:

- Audio recorder
- Consent form (send via email/post prior to interview if telephone interview)
- PIS (send via email/post prior to interview if telephone interview)

RESEARCH AIM

What were recruited research site staff's perceptions of the recruitment process and how did research sites deal with recruiting to two large randomised trials?

1) Welcome and context setting:

- Introduce yourself
- Guide the participant through the Participant Information Sheet
- "The interview will last approximately 20 minutes"
- Explain that, "to help us with this study, we would like to make a recording of what we all say today, but nobody will be able to identify you from that recording other than me. Is that okay?"
- Guide the participant through the consent form. If happy to participant, ask to sign (if interview is being undertaken remotely then guide participant through audio consent process).
- "You are free to withdraw at any point and you don't have to answer any of the questions if you don't want to."
- Check they are happy to continue and ask if there are any questions.

2) Proceed to interview:

Question 1: How did you undertake recruitment to the endometrial scratch trial?

Prompts:
How did you decide who to approach?
When did you approach potential participants?
How did you approach potential participants?

Question 2: How did you find explaining randomisation, and the outcome of randomisation, to trial participants?

Prompts:
How did you explain randomisation?
Did you encounter any challenges in explaining randomisation?
How did you find participants comprehension of randomisation?

How did you experience informing participants they were randomised to the control arm?
Did of any your participants have a negative response/reaction to being randomised to the control arm? How did you deal with this?

Question 3: How did you find the recruitment materials? Did you need to supplement these with any additional information?

Prompts:
How did you find the trial PIS/consent form/video in informing the participant about the trial and then ES?
Did you provide the participant with any additional information regarding ES not contained within the PIS/consent form/video? Did they signpost the participants to any other resources?
How did you deal with participants who struggled to understand key aspects of the trial (e.g. randomisation or intervention)
Did you use pictures/diagrams?
Did you provide participants with any additional details in order to aid their understanding/decision to participate in the trial?

Question 4: What were the challenges you encountered over the course of the trial?

Prompts:
Do you think your IVF unit was suitably set up to run this trial? (knowledge and skill level) Did you have capacity to run the current trial? (resources) Would you have capacity to integrate further trials at the same time?

Question 5: Did recruitment practices or your opinion of the intervention change over the course of the trial?

Prompts:
Did you or did you not have any preference for the endometrial scratch for improving success rates? What are your current opinions on the ES? How has the changed since the start of the trial?
How did newly published research during the course of the trial affect your recruitment practices and your opinion of the ES?
Did participants enquire about the new research? Both those newly screened and already in the trial?

Question 6: How did you discuss the endometrial scratch procedure with participants?

Prompts:
Did you encounter any issues with participants concerned that participation in the trial would cause delays to IVF? How did you deal with this?
How did you discuss recovery from the procedure?
Did participants enquire regarding opportunities to receive ES outside of the trial? How did you deal with this?
Did you inform participant whether or not they could receive ES outside of the trial?

Question 7: Prior to taking part in the trial, did participants have any expectations or preconceptions of ES?

Prompts:

Did participants have any knowledge of ES at the time of entering the study?

How did you manage those expectations?

Did you notice that the participant had any positive or negative preconceptions?

Question 8: How did you find your site managed the scheduling of the ES procedure?

Question 9: Did any of your participants decline the ES? How did you deal with this?

Prompts:

If participants were unable to attend an initial ES procedure, how did you respond?

Question 10: How did you manage participants pain during and post ES procedure?

Prompts:

What support did you provide particpants post procedure?

Did pariticipants believe that any post procedure phenomena were linked to ES?

Was your centre recruiting to more than one trial at once?

IF YES: Question 11: How did you find recruiting to multiple studies? How did you chose which participant to approach each trial?

Prompts:

Did you use the joint information sheets and letters?

Did you have any systems set up in order to recruit to both trials?

What do you think that patients thought about being approached to two large randomised trials?

Question 12: How did you manage negative pregnancy outcomes? Did participants present with any concerns that their randomisation outcome affected the success of their IVF?

Question 13: How did you find using the database and communicating with the central trial management team?

Prompts:

Weekly emails – too much information? Database – any ways of making more user friendly? Would prefer more email/phone/in person contact?
