# Supplementary material for: Experiences of trial participants and site staff of participating in and running a large randomised trial within fertility (the endometrial scratch trial): a qualitative interview study
Source: BMJ Open. 2021 Sep 16;11(9):e051698. doi: 10.1136/bmjopen-2021-051698 (PMC8449983; doi:10.1136/bmjopen-2021-051698)
Supplement: Supplementary data [file bmjopen-2021-051698supp001.pdf]

Endometrial Scratch Trial Participant Topic Guide

NOTES: This topic guide is a flexible tool and may be revised as new areas of interest arise during the process of data collection. The wording of questions is for guidance only and can be varied to suit the natural style of the interviewer and the level of understanding of the participant.

ITEMS REQUIRED:

- Audio recorder
- Copies of questionnaires and text messages (or send over to participant via email/post if interview to be undertaken over the phone).
- Consent form (returned via post before interview, or audio recording of consent form undertaken prior to interview)
- PIS (send via email/post prior to interview if telephone interview)
- Look to see if participant received and replied to text messages, paper and/or electronic questionnaires.

RESEARCH AIM

1. What was the reason for participants withdrawing from the intervention?
2. What were recruited trial participant's and research site staff's perceptions of the recruitment process and how did research sites deal with recruiting to two large randomised trials?
3. What were recruited participant's perceptions of the data collection tools used during the trial, specifically, text messages to collect pain and electronic questionnaires?

1) Welcome and context setting:

- Introduce yourself
- Guide the participant through the Participant Information Sheet
- "The interview will last approximately 30 minutes"
- Explain that, "to help us with this study, we would like to make a recording of what we all say today, but nobody will be able to identify you from that recording other than me. Is that okay?"
- Guide the participant through the consent form. Take audio recording of responses (if consent form hasn't been returned already by post)
- "You are free to withdraw at any point and you don't have to answer any of the questions if you don't want to."
- Check they are happy to continue and ask if there are any questions.

2) Proceed to interview:

Research questions (RQ) with associated opening participant questions (PQ) and participant prompts (PP)

Recommended general prompts (Note: list is not exhaustive):

- *Can you please elaborate?*
- *Tell me more.*
- *Could you provide an example please?*

Endometrial Scratch Participant Topic Guide V1.0 dated 26/11/2018

FOR ALL PARTICIPANTS:

RQ3: What were recruited trial participant's perceptions of the recruitment process and how did research sites deal with recruiting to two large randomised trials?

PQ1: *How were you approached to take part in the Endometrial SCRATCH trial?*

PPa: *How did you feel about the way that you were approached? How was the study introduced to youI? Is there anything that we can do to improve the recruitment process?*

PPb: Do you remember receiving any information about the trial? **If yes,** *how do you feel about the information you had to read about the trial?*

PPc: *If you were to take part again, what information would you need to make the decision to participate? What type of information would be helpful to you?*

PPd: *Why did you choose to take part in the study?*

PPe: *How did you feel about being involved in research while undergoing IVF?*

PPf: *Did you have any preconceptions about the Endometrial Scratch procedure prior to taking part in the trial (i.e. its positive/negative effect)? Do you know where these preconceptions were from?*

PQ2: *Were you approached about any other trials?*

IF YES, PQ3: *How did you find being approached regarding more than one trial?*

PPa: *Did you receive the joint information sheet/letter explaining both e-Freeze and Endometrial Scratch trials? Did you find it useful in informing your decision to participate?*

PPb: *How was the trial/how were the trials introduced to you?*

PPc: *How did you decide which trial to participate in?*

FOR TAU PARTICIPANTS

How did you feel about being randomised to not receive the scratch? (If participant describes feeling of disappointment, sadness, etc.) *Did you speak to staff about your feelings about the outcome of the randomization? How did the staff respond to you? (Did you feel supported? Adequately informed?)*

FOR PARTICIPANTS WHO CHOSE NOT **TO RECEIVE THE INTERVENTION:**

RQ 1: What was the reason for participants withdrawing from the intervention?

PQ4: *Our records show that you chose not to receive the endometrial scratch – can you provide some insight into this please?*

PPa: *Did anything about the way the procedure was introduced to you, or the way it was arranged, influence your decision?*

PPb: *Can you explain some of the factors that influenced your decision?*

PPc: *How did you feel when you discussed your decision with staff at the IVF unit/clinic?* (note: try and find out if staff could have done any more to facilitate delivery of ES)

FOR PARTICIPANTS WHO RECEIVED THE INTERVENTION:

RQ2: Of those women who did receive the intervention, what were their thoughts of the procedure and the information they received in preparation for the procedure?

PQ6: *How did you find receiving the endometrial scratch?*

PPa: *Would you or would you not have it again? How come?*

PPb: *What did you think of the written/verbal information you received from the fertility unit prior to having the endometrial scratch?*

PPc: *What would you say to other people that are interested in receiving the ES procedure?*

PPd: *How did you feel about your preparedness to receive the procedure? What would have helped with your preparedness, in your opinion?*

PPe: *Should other people receive the procedure?*

FOR ALL PARTICIPANTS:

RQ4: What were recruited participant's perceptions of the data collection tools used during the trial, specifically, text messages to collect pain and electronic questionnaires?

PQ8: *I would now like to ask about the questionnaires and text messages we have sent you to collect information. What did you think of these?*

ONLY THOSE WHO RECEIVED THE INTERVENTION: PPa: *How did you find the pain score text messages that were sent to you?*

PPb: *You received an electronic questionnaire. How did you find completing this? Could it be improved?*

PPc: *Would you rather receive an electronic questionnaire or paper questionnaire?*

PPd: *Which is your preferred way to collect information from you? Electronic or paper?*

Endometrial Scratch Participant Topic Guide V1.0 dated 26/11/2018
