# Supplementary material for: Experiences of trial participants and site staff of participating in and running a large randomised trial within fertility (the endometrial scratch trial): a qualitative interview study
Source: BMJ Open. 2021 Sep 16;11(9):e051698. doi: 10.1136/bmjopen-2021-051698 (PMC8449983; doi:10.1136/bmjopen-2021-051698)
Supplement: Author's manuscript [file bmjopen-2021-051698.draft_revisions.pdf]

BMJ Open is committed to open peer review. As part of this commitment we make the peer review history of every article we publish publicly available.

When an article is published we post the peer reviewers' comments and the authors' responses online. We also post the versions of the paper that were used during peer review. These are the versions that the peer review comments apply to.

The versions of the paper that follow are the versions that were submitted during the peer review process. They are not the versions of record or the final published versions. They should not be cited or distributed as the published version of this manuscript.

BMJ Open is an open access journal and the full, final, typeset and author-corrected version of record of the manuscript is available on our site with no access controls, subscription charges or pay-per-view fees (http://bmjopen.bmj.com).

If you have any questions on BMJ Open's open peer review process please email info.bmjopen@bmj.com

BMJ Open

The experiences of trial participants and site staff of participating in and running a large randomised trial within fertility (the Endometrial Scratch Trial) – a qualitative interview study

Journal:	*BMJ Open*
Manuscript ID	bmjopen-2021-051698
Article Type:	Original research
Date Submitted by the Author:	25-Mar-2021
Complete List of Authors:	Chatters, Robin; The University of Sheffield, Clinical Trials Research Unit, School of Health and Related Research<br>White, David; The University of Sheffield, Clinical Trials Research Unit<br>Pye, Clare; Sheffield Teaching Hospitals NHS Foundation Trust, Jessop Wing<br>Petrovic, Ana; Rotherham Doncaster and South Humber Mental Health NHS Foundation Trust<br>Sizer, Anya; Fertility Network UK<br>Kumar, Pavithra; The University of Sheffield, Clinical Trials Research Unit<br>Metwally, Mostafa; Sheffield Teaching Hospitals NHS Foundation Trust
Keywords:	Clinical trials < THERAPEUTICS, Reproductive medicine < GYNAECOLOGY, QUALITATIVE RESEARCH

SCHOLARONE™
Manuscripts

I, the Submitting Author has the right to grant and does grant on behalf of all authors of the Work (as defined in the below author licence), an exclusive licence and/or a non-exclusive licence for contributions from authors who are: i) UK Crown employees; ii) where BMJ has agreed a CC-BY licence shall apply, and/or iii) in accordance with the terms applicable for US Federal Government officers or employees acting as part of their official duties; on a worldwide, perpetual, irrevocable, royalty-free basis to BMJ Publishing Group Ltd ("BMJ") its licensees and where the relevant Journal is co-owned by BMJ to the co-owners of the Journal, to publish the Work in this journal and any other BMJ products and to exploit all rights, as set out in our licence.

The Submitting Author accepts and understands that any supply made under these terms is made by BMJ to the Submitting Author unless you are acting as an employee on behalf of your employer or a postgraduate student of an affiliated institution which is paying any applicable article publishing charge ("APC") for Open Access articles. Where the Submitting Author wishes to make the Work available on an Open Access basis (and intends to pay the relevant APC), the terms of reuse of such Open Access shall be governed by a Creative Commons licence – details of these licences and which Creative Commons licence will apply to this Work are set out in our licence referred to above.

Other than as permitted in any relevant BMJ Author's Self Archiving Policies, I confirm this Work has not been accepted for publication elsewhere, is not being considered for publication elsewhere and does not duplicate material already published. I confirm all authors consent to publication of this Work and authorise the granting of this licence.

For peer review only

Title: The experiences of trial participants and site staff of participating in and running a large randomised trial within fertility (the Endometrial Scratch Trial) – a qualitative interview study

Robin Chatters (corresponding author) – Sheffield Clinical Trials Research Unit, School of Health and Related Research, The University of Sheffield. R.chatters@sheffield.ac.uk

David White - Sheffield Clinical Trials Research Unit, School of Health and Related Research, The University of Sheffield. d.a.white@sheffield.ac.uk

Clare Pye – Assisted Conception Unit, Jessop Wing, Sheffield Teaching Hospitals NHS Foundation Trust. clare.pye1@nhs.net

Ana Petrovic – Rotherham, Doncaster and South Humber NHS Foundation Trust. ana.petrovic@nhs.net

Anya Sizer – Fertility UK. anya@fertilitynetworkuk.org

Pavithra Kumar - Sheffield Clinical Trials Research Unit, School of Health and Related Research, The University of Sheffield. p.kumar@sheffield.ac.uk

Mostafa Metwally - Assisted Conception Unit, Jessop Wing, Sheffield Teaching Hospitals NHS Foundation Trust. m.metwally@sheffield.ac.uk

Word count: 4086 words

For peer review only

For peer review only - http://bmjopen.bmj.com/site/about/guidelines.xhtml

ABSTRACT

Objectives: To explore the experiences of endometrial scratch (ES) trial participants and site staff of trial recruitment and participation, in order to improve the experience of participants in future trials.

Design: Qualitative study of a subset of participants in the ES randomised controlled trial (RCT), and a subset of trial site staff.

Setting: A purposeful sample of nine of the 16 UK Fertility Units that participated in the trial.

Participants: A purposeful sample of 27 trial participants and seven site staff.

Results: Participants were largely happy with the recruitment practices, however, some were overwhelmed with the amount of information received. Interviewees had positive preconceptions regarding the possible effect of the ES on the outcome of their IVF cycle, which often originated from their own internet research and seemed to be exacerbated by how site staff described the intervention. Some participants appeared to not understand that receiving the ES could potentially reduce their chances of a successful IVF outcome. Those randomised to the control arm discussed feeling discontent; site staff developed mechanisms of dealing with this.

Conclusions: A lack of equipoise in both study participants and the recruiting site staff led to trial participants having positive preconceptions of the potential impact of the ES on their upcoming IVF cycle. Trial participants may not have understood the potential harms of participating in a randomised trial. The trial information sheet did not clearly state this; further research should assess how such information should be presented to potential participants, to proportionately present the level of risk, but to not unduly discourage participation. The amount of information fertility patients require about a research study should also be investigated, in order to avoid participants feeling overwhelmed by the amount of information they receive prior to starting IVF.

Trial registration: ISRCTN, ISRCTN23800982, post results.

KEYWORDS

Clinical trial; reproductive medicine; qualitative research.

STRENGTH AND LIMITATIONS OF THIS STUDY

- We present data from a purposeful sample of 27 trial participants and seven site staff regarding their experiences of taking part in, and running, the endometrial scratch randomised controlled trial.
- As far as we are aware, this is the first study to investigate the experience of fertility patients of being recruited to, and participating in, a randomised trial.
- We included both participants randomised to the control and intervention arms of the trial, plus those who did and did not have a live birth after their IVF cycle.
- Recall may be an issue, as trial participants were interviewed on average 17.8 months after they were recruited to the trial.
- We only interviewed individuals who participated in the trial, and therefore interviews may represent a biased view of the experiences of being recruited to and participating in the trial.

INTRODUCTION

A wealth of literature exists that qualitatively assesses the experiences of participants being recruited to trials across various disease areas, indicating that participants can find it difficult to

For peer review only - http://bmjopen.bmj.com/site/about/guidelines.xhtml

understand the potential harms of participating in a study [1,2], and staff experience challenges in expressing equipoise to participants [3]. The participant information sheet (PIS) has been extensively researched; in one study the 'traditional' PIS was found to oversupply information to 79.5% of participants [4]. Other studies have identified that, if given a choice, participants often opt for a shorter version of the PIS [5,6]. To our knowledge, the experience of patients participating in randomised trials in fertility has not been explored, although recruitment techniques have been described across two trials [7].

The Endometrial Scratch Trial was a randomised controlled trial (RCT) that aimed to assess the clinical and cost effectiveness of undertaking the endometrial scratch prior to first time in vitro fertilisation (IVF). Participants (women recruited between July 2016 and October 2018 from 16 fertility centres across the UK) were randomly allocated to either receive usual IVF treatment, or the endometrial scratch (ES) procedure, followed by usual IVF treatment. The ES involves scratching the lining of the womb (the endometrium) prior to a cycle of IVF and is hypothesised to improve the chances of the embryo implanting by improving the receptivity of the endometrium [8]. A lack of good quality evidence exists to support the delivery of the ES in women undergoing their first cycle of IVF [9]. The full trial protocol is published elsewhere [10].

There are several reasons to investigate the experience of participants and staff within this fertility trial. Undergoing IVF can be a distressing and emotional experience that involves patients receiving invasive procedures, causing both physical and mental stress [11,12]. Patients receive a lot of information prior to their treatment, with increased amounts of information being associated with anxiety [13]. It is therefore important to ensure that participation in research does not create an extra burden for the patient. Furthermore, as women undergoing fertility treatments often seek information from sources other than their fertility team (e.g. the internet, books and magazines), it is pertinent to ascertain how this affected the participant's experience of recruitment to the trial [14]. The study involved potentially difficult decisions for participants, specifically, whether to participate in the trial given the 50% chance of being randomised to a novel intervention with unknown benefits or harms, or the treatment as usual control group. Qualitative evaluations in other trial populations have identified that participants often feel discontent when randomised to the treatment as usual arm [15]. Given that recruiting staff have been found to struggle to convey equipoise in previous studies [3,16], it is interesting to evaluate if these individuals conveyed equipoise, and if this impacted on the participant's experiences of the trial.

The aim of this qualitative study was to understand the experiences of participants being recruited to - and participating in – the endometrial scratch trial, including any issues or barriers they faced. We also aimed to understand trial site staff's experiences, with a general aim to improve the experience for participants in future trials.

METHODS

Sampling, approach and recruitment

Purposive sampling was used to achieve the maximum variation in characteristics across trial participants and staff. Firstly, six trial sites were selected, by seeking distribution of the characteristics described in Table 1 (site sampling attributes). Trial participants who had recently completed the trial and had not withdrawn from trial follow-up were then sampled from these sites, using the attributes presented in Table 1 (participant sampling attributes).

Table 1: Site and participant level sampling attributes

Attribute	Categories
**Site sampling attributes**	

For peer review only - http://bmjopen.bmj.com/site/about/guidelines.xhtml

Site type	NHS site/privately owned
Size of centre	number of IVF cycles undertaken per year
Consent rate of centre	the proportion consented relative to those screened
**Participant sampling attributes**	
Intervention status	ES received, ES declined, treatment as usual
Age	30 or below, above 30
Duration of infertility	Less than 36 months, 36 months or more
Outcome of first cycle of IVF	Live birth, no live birth

Site staff (principle investigators and research nurses) from the six selected centres were initially sampled for the staff interviews. Due to low staff availability and willingness to participate in the interviews, additional sites were selected as long as the research nurse was still in post and had undertaken recruitment to the trial.

Potential interviewees were initially approached by letter or email. If no contact was received, individuals were telephoned or emailed approximately one week after the initial contact was made. Consent was obtained immediately prior to the interview via telephone, with the conversation regarding consent being audio recorded.

Research team and reflexivity

One woman (AP, research assistant with a PhD) and one man (RC, trial manager with a BSc) conducted the interviews with all participants. Both had prior training and experience of undertaking qualitative interviews.

RC had managed the Endometrial Scratch Trial from the start; AP commenced her involvement more recently, just prior to the start of this qualitative study. A relationship between site staff and RC was therefore already formed, which was also true for AP but to a lesser extent. Site staff would have been aware of the interviewer's involvement in the design and delivery of the trial and their reasons for doing the research.

The interviewers had no involvement in the clinical care of the trial participants, nor had they had previous contact. Participants would have been aware of the interviewer's reasons for undertaking the research and may or may not have been aware of the interviewers' involvement in the trial in which they participated.

Data collection

A semi-structured interview schedule was used to guide the interviews – with separate schedules being used for trial participants and site staff. The schedules were pilot tested in one interview per schedule, and adjustments were made following discussions between AP and RC; the pilot interview is included in the analysis. The participant interviews were undertaken prior to the staff interviews in order to allow themes from the former to be discussed with staff. Prompts were used during interviews and repeat interviews were not carried out.

For peer review only - http://bmjopen.bmj.com/site/about/guidelines.xhtml

Interviews were audio recorded and transcribed verbatim. There were no other individuals present at the interviews, except for, in a few instances, the participant's child. The interviewee recorded notes after each interview. Interviews with trial participants ranged from 4.5 to 21.5 minutes, and those with staff were 19.5 to 57.6 minutes.

The trial participant interview schedule covered the participants' experience of recruitment and participation in the trial. The staff interview schedule included the staff's experience of recruitment and their overall experience of delivering the trial at their site.

All interviews were undertaken by telephone. Data saturation was discussed between RC and AP separately for staff and trial participant interviews.

Data analysis

An inductive thematic analysis was used to analyse the data, which was undertaken within a phenomenological framework. We used Braun and Clark's reflexive thematic analysis framework, which involves six phases of analysis—familiarisation, initial coding, theme construction, reviewing themes, defining themes and producing the report [17]. Two researchers (AP and RC for the trial participant interviews, and PK and RC for the staff interviews) developed the coding structure separately by coding a small number of transcripts (four for the participant interviews and two for the staff interviews). NVivo (v12) was used to undertake all analyses. The researchers then met and resolved any differences in coding in order to develop the final list of codes. All other transcripts were then coded by one researcher, either AP or RC for the trial participant interviews, and RC or PK for the staff interviews. The coding trees were formed of overarching themes that were related to the overall aims of this qualitative study - the experience of receiving/delivering ES, reasons for participants deciding not to receive ES after being randomised to do so, preconceptions of the ES procedure, and experiences of recruitment. However, in this manuscript, only the latter two themes are reported in order to reflect those themes that have the potential to improve the experience of participants in future fertility studies. RC undertook all analysis steps after coding, including formation of themes and writing of the report. Interviewees did not feedback on the findings or transcripts.

Patient and public involvement

Input into the design of the study, interview schedules and patient facing materials were sought from the Sheffield Reproductive Health Research Public Advisory Panel and the trial's public involvement representative (AS), who also reviewed the results of the study.

Ethics

This qualitative substudy was approved by Berkshire South Central REC.

RESULTS

Interviews and characteristics of participants

Forty-four trial participants were approached to participate in the study, of which two actively declined to participate and 15 either did not respond or were not contactable. As a result, 27 trial participants took part.

Interviewed participants had an average duration of infertility of three years (range 11 months to 8 years 6 months) and were interviewed, on average, 17.8 months after they were randomised into the trial (range from eight to 33 months). The demographics of the trial participants that took part in this study are summarised in *Table 3*.

Table 3. Characteristics of interviewed trial participants

ES, endometrial scratch; ID, identification number; N/A, not applicable; TAU, treatment as usual

For peer review only - http://bmjopen.bmj.com/site/about/guidelines.xhtml

For peer review only

Seven site staff (one principle investigator (PI) and six research nurses) took part. Fifteen participants were invited (seven PIs and eight research nurses) – one participant actively declined due to lack of time, five participants did not reply to initial contact attempts, and interviews were attempted to be scheduled for two individuals but a mutually agreed time could not be found. The characteristics of the participating site staff can be found in *Table 4*.

Table 4: Characteristics of interviewed site staff

NHS, National Health Service; PI, Principle Investigator; RN, Research Nurse

Trial participants and site staff discussed three major themes during the interviews – the experience of recruitment to the trial, the written materials that were provided at recruitment, and the lack of equipoise in both trial participants and site staff and the demoralising effect this had on some participants randomised to the control arm. Below, we discuss these themes.

Experience of the general recruitment process

Trial participants were generally happy with the recruitment process.

> *"I think it was all done really well. It was just, it was really well explained and I don't … think, if it was happening again, … I wouldn't change anything about it"* Participant 2 – Site 8

Participating in research whilst undergoing IVF was seen by many as "exciting" and "interesting", and provided an alternative focus for two participants.

> *"I think it's in a way it was actually it was quite a good thing because I think it gave me something else to focus on […] it was quite nice to have something that was being discussed where I felt actually this has got a wider benefit beyond just what's happening with me and my husband"* Participant 4 – Site 4

Three participants felt that the approach to take part in the trial was too informal, with the participants either having to ask the site staff about the trial, or the trial being mentioned to the participant at the last minute when there were previous opportunities to raise this.

> *"I don't know if it should be more formal really, because it was just kind of, if they remembered on that day that I would be a, I'd be a possible candidate so it wasn't, do you know what I mean, maybe you should get it in a pack or something at the beginning, or told at the very beginning."* Participant 2 – Site 1

Written information provided at recruitment

Four participants could not recall the recruitment materials, due to the time that had elapsed since they were recruited. Of those that could, five participants stated that the materials were clear and informative.

> *"I thought the information sheet was really useful and informative."* Participant 4 – Site 7

There were differences in opinion with regards to the amount of information that trial participants were asked to read during the recruitment process, with some participants stating that the materials were not too onerous to read.

> *"Yeah I think it was maybe like a double sided page it wasn't kind of too much."* Participant 2 – Site 2

However, others reflected on the amount of information provided more negatively, with two

For peer review only - http://bmjopen.bmj.com/site/about/guidelines.xhtml

participants discussing the amount of information they received as “overwhelming”.

> *“it can be very overwhelming, having lots of trial information given to you at the same time as all your IVF treatment. It just means that your pack of information to read through, I think I had about twenty different documents or leaflets”* Participant 4 – Site 7

One participant suggested that the PIS should contain more information about related studies.

> *“After reading about other studies in the newspapers like The Guardian and things like that, it might have been also interesting to hear a little bit about other studies that were going on… Because, I think when I did read about this study in The Guardian…and it suggested that it wasn’t helpful. There was part of me that I think became a little bit angry that maybe I’d put myself through it at the time.”* Participant 2 – Site 7

Equipoise

Participant’s lack of equipoise were exemplified by eighteen of the interviewed participants stating that a major reason to participate in the trial was to increase their chances of a positive outcome from their IVF cycle.

> *“obviously if they’d have said to us the Scratch trial probably won’t have improved our chances of getting pregnant, I probably wouldn’t have, wouldn’t have taken part. So, we, you know we did it on the basis that we wanted to be on the clinical sample so that we could improve our chances of getting pregnant.”* Participant 1 – Site 7

There seemed to be a lack of understanding of the potential for the ES to negatively affect the changes of live birth, with five trial participants believing that the ES procedure could not “harm” them.

> *“There wasn’t anything that could potentially harm my chances of success. It wasn’t, you know, it wasn’t like trying out a new medicine or anything like that”* Participant 2 – Site 8

Source of positive preconceptions

Trial participants discussed that a small number of site staff supported their positive preconceptions, sometimes in a subtle way.

> *“You don’t know whether it helps or not but there seems to be this, this influence from some within the clinic that almost nudge nudge, wink wink, you know,’ you’re gonna be fine because this really does help.”* Participant 2 – Site 7

In other cases, the trial participant reported that they believed site staff thought ES would increase their changes of a live birth

> *“basically they said: “It’s completely up to you, it’s something that we’re giving to you, you can decide.” … obviously they do believe scratches increase chances of success.”* Participant 3 – Site 7

However, for some participants, the information provided by site staff ensured they were aware that there was insufficient evidence to suggest that the procedure will improve their chances of success.

For peer review only

For peer review only - http://bmjopen.bmj.com/site/about/guidelines.xhtml

> *"The consultant suggested that there wasn't much evidence about whether the scratch was helpful or not when it comes to success of IVF."* Participant 1 – Site 8

Participants also sought information from other sources, most frequently from the internet. For five participants, the internet provided the participant with positive preconceptions of the effect of the ES on their upcoming IVF cycle.

> *"Just the success rate for the people who were trying to do IVF; who were trying to have babies. The success rate after the scratch was high."* Participant 2 – Site 5

However, for two participants, reading information on the internet led them to a position of equipoise.

> *"I wasn't sure, cus some studies said it was successful, others said it wasn't, so I was on the fence"* Participant 2 – Site 1

Discontent when randomised to the control arm of the trial

Positive preconceptions of the effect of the ES on the outcome of their IVF cycle led five out of the eight interviewed participants randomised to the control arm feeling some level of discontent with the outcome of randomisation. Some participants discussed feeling slight disappointment.

> *"I think there was a slight bit of disappointment which is ridiculous really because it's randomized. I wasn't sort of like devastated or anything like that... At the end of the day I was having the treatment. It was, that's what current practice is, to not have the scratch for a first time if under 35 so I was just like well we'll go with that and see what happens. So I didn't dwell on it or anything like that."* Participant 4 – Site 7

For others, randomisation to the control group triggered significant demoralisation.

> *"I was really quite disappointed and quite emotional when I, didn't get on the, Scratch trial itself because I was worried that because I didn't have that, that my chances of getting pregnant were minimized"* Participant 1 – Site 7

Staff awareness of positive preconceptions and demoralisation

Site staff were aware of the trial participant's positive preconceptions.

> *"They would generally say 'oh I've heard that, about the procedure and it can benefit the chances of you getting pregnant'."* RN-SITE4

Site staff felt the need to manage the participant's expectations in order to prevent resentful demoralisation upon randomisation to the control arm.

> if they are going, to really want a Scratch but sign up anyway and then sneak off and get one privately and not let me know [yes] then that's not good for the research. RN-Site9

In order to attempt to dispel any positive preconceptions during the recruitment process, site staff spent time explaining the lack of an evidence base for ES, and the need for a RCT, to potential trial participants.

> *"we did explain to them comprehensively the evidence, around the Endometrial Scratch and the requirement for a big trial like this to be conducted for us to be able to know the answers and to know whether they should be offered to all patients going through forward for IVF."* PI-SITE1

For peer review only - http://bmjopen.bmj.com/site/about/guidelines.xhtml

However, site staff reported that participants seemed to struggle to understand the need for a control arm when some studies reported a benefit to having the ES.

> *"Sometimes patients found it difficult to accept that if there has been some study that indicate potential benefit, then patients found it difficult to accept that they could be randomised into the control arm."* PI-SITE1

Many site staff were also aware of some participant's disappointment of being randomised to the control arm.

> *"Some people did express great disappointment and in particularly that way round actually it was nearly always the ones that had wanted it obviously cos it's the intervention."* RN-SITE6

Staff described mechanisms they had developed for dealing with this disappointment, summarised in table 5.

Table 5 – methods site staff used to deal with participant's disappointment at being randomised to the control arm of the trial.

**Method of dealing with disappointment**	**Quote**
Explain the importance of the control group	*"So I think its explaining the importance of being in that control group to them. And how we can, you know, we don't know if there was any benefit until we've actually, that's the whole purpose of doing the trial, to answer a question."* RN-SITE1
Describe the lack of evidence to support ES	*"Try and sort of explain that, there was no evidence whether it was a benefit anyway, and that normally if they were going through their treatment, I understand they wouldn't be having a Scratch, so it wasn't as if they missing out on anything."* RN-SITE6
Explain the increased contact with staff that participating in research allows	*"If it's the control you know it's a bit of extra contact with people and I would always say 'look I'm here in the unit, you're not having the Scratch, but if I can help you in any other way, feel free to ring the research mobile. You know to try and always make it I suppose to soften the blow really, and for those who were disappointed."* RN-SITE9

DISCUSSION AND IMPLICATIONS

Main findings

This qualitative study demonstrates that participants recruited to the Endometrial Scratch Trial were, on the whole, happy with the recruitment practices utilised in the trial. However, a small number of participants felt that the invitation to participate in the trial was too informal, and a minority described the amount of written material they received as "overwhelming". There appeared to be a lack of equipoise amongst both sets of interviewees, with regards to the potential effect of the ES on the outcome of the upcoming IVF cycle. Trial participants had positive preconceptions of the ES

For peer review only - http://bmjopen.bmj.com/site/about/guidelines.xhtml

procedure, which often originated from the trial participant's own internet research, and in some instances, participants indicated that they believed site staff supported these preconceptions. Some trial participants appeared to not be aware that receiving the ES could potentially reduce their chances of a successful IVF outcome. This, coupled with the positive preconceptions of the ES, led to dissatisfaction amongst some participants randomised to the control arm.

Strengths and limitations

A strength of this study is that it included a large number of women that participated in a large RCT. Participants who were randomised to both the control and intervention arms, as well as those participants whose IVF cycle did and did not result in a live birth, were interviewed in order to minimise this possible bias on their reflections of their experience. We also interviewed staff in order to seek their experiences. A limitation includes that only those that participated in the trial were interviewed, with those participants that withdrew from the trial not being interviewed. This may result in a skewed reflection of the experience of recruitment to the trial. In addition, participants were interviewed, on average, 17.8 months after they agreed to participate in the trial, and furthermore, were interviewed after their IVF cycle had completed. This therefore results in participants reflections on their experiences being clouded by time, and being affected by the outcome of their IVF cycle.

Comparison with existing literature

Previous studies have identified that potential participants have different information needs, and may require different versions of the PIS [5,6]. Our findings support this, with some participants reporting to be "overwhelmed" by the information they received, whilst others thought that is was the right amount. Undergoing IVF can be a physically, emotionally and financially challenging process; Information provided to patients at the start of an IVF cycle can overwhelm participants and cause anxiety [13]. This highlights a particular challenge within IVF research of conveying the necessary information to participants in order to allow informed consent.

Our study mirrors the findings of other studies that have described the discontent felt by participants when randomised to the control group of an RCT [18,19], participants seeking information from internet sources [14], and the motivation to receive the intervention has been reported as a major reason participants decide to take part in interventional trials [20,21]. In this study, site staff tried to reposition the participant's positive preconceptions of the ES by explaining the evidence base, which has also been reported in other studies [15,20]. However, it appears that in some cases, participants received cues from site staff that the ES may benefit them, despite having no evidence to support this. Staff have been found to communicate equipoise poorly in other studies, including providing inbalanced descriptions of trial treatments and predicting RCT outcomes [16].

Other studies have also reported that participants overestimate the benefits, and underestimate the risks, of participating in research [1,2]. Our study adds to this evidence base by reporting that, in our study, participants 'arrived' with positive preconceptions, which, in the most part, originated from research undertaken on the internet. This, coupled with subtle signals from recruiting site staff, led to positive preconceptions of the potential effect of the ES procedure on their IVF outcome, and potentially an underestimation of the potential harms of the procedure. This may have been exacerbated by the time-sensitive nature of fertility treatment and the desire for a positive outcome; both clinicians and patients have been reported to feel the need to improve the outcomes of treatment immediately following a failed treatment cycle [22].

Implications

When recruiting participants to randomised trials in fertility, recruiting staff should be aware that participants may base their decision to participate on internet research, which risks participants making their own subjective interpretation of the evidence, and may result in positive preconceptions of the potential benefits - and a downplaying of the potential harms - of receiving

For peer review only - http://bmjopen.bmj.com/site/about/guidelines.xhtml

the novel intervention. As such, it seems that some participants require more detailed information about the research literature than was provide in the Endometrial Scratch trial PIS. Other methods of providing such information to potential participants should be explored, possibly by providing participants with a choice of levels of information, as suggested in previous studies [6,26].

Recruiting staff should try to introduce the control group with similar emphasis to the intervention group, however, discontent once randomised to the control group discontent may still be felt, in which case we suggest three ways of lessening this (see table 5). Other studies have provided recommendations to guide 'recruitment conversations' in surgical trials – these guidelines are also applicable to studies in fertility [23–25].

Future research

Future research should focus on how the potential harms of taking part in a research study can be relayed to participants, in order to deal with overly positive preconceptions and the impact of these preconceptions on feeling discontent when randomised to the control arm of the trial. The PIS for the Endometrial Scratch Trial did not explicitly state the evidence for or against the endometrial scratch procedure, nor did it explicitly state the ES may potentially harm the chances of a successful IVF outcome. However, future research should seek trial participant's views on how evidence regarding the potential harms of a procedure, or at least the unknowns, should be imparted, including the medium (i.e. PIS, or face to face) and how the information should be structured in order to not unnecessarily perturb potential participants from participating in research. Given that research undertaken in other populations suggests participants prefer shorter information sheets [6], and that participants only minimally use the PIS [27], patient acceptable alternatives to the patient information sheet should be investigated, in order to reduce the burden on patients at the start of their fertility treatment.

Footnotes

Acknowledgements: We would like to thank those trial participants and site staff that gave their time and participated in the qualitative interviews.

We would also like to thank the Reproductive Health Research Public Advisory Panel at the Jessop Wing, Sheffield Teaching Hospital NHS Foundation Trust, for their input into the design of this qualitative study.

Contributors: RC designed the study and led on data collection, analysis and the first draft of the manuscript. DW and CP provided input into the design of the study, analysis and reviewed and approved the manuscript. AS provided input from a lay-person perspective into the design of the study, patient facing materials, results and manuscript. AP inputted into the design of the study, assisted with data collection and analysis, and reviewed and approved the final version of the manuscript. PK assisted with data analysis, and reviewed and approved the final version of the manuscript. MM inputted into the design of the study and reviewed and approved the final version of the manuscript.

Data availability statement: No data are available. The experiences expressed during the interviews refer to sensitive and personally relevant stories; it was agreed that transcripts would not be shared with other researchers in order to preserve the anonymity of study participants.

Competing interests: None declared.

Patient consent for publication: Not required.

For peer review only - http://bmjopen.bmj.com/site/about/guidelines.xhtml

Funding: This study is funded by the National Institute for Health Research (NIHR) [Health Technology Assessment Programme (project reference 14/08/45)]. The views expressed are those of the author(s) and not necessarily those of the NIHR or the Department of Health and AQ5 Social Care.

REFERENCES

1 Lidz CW, Appelbaum PS, Grisso T, *et al.* Therapeutic misconception and the appreciation of risks in clinical trials. *Soc Sci Med* 2004;**58**:1689–97. doi:10.1016/S0277-9536(03)00338-1

2 Canvin K, Jacoby A. Duty, desire or indifference? A qualitative study of patient decisions about recruitment to an epilepsy treatment trial. *Trials* 2006;**7**:1–13. doi:10.1186/1745-6215-7-32

3 Phelps EE, Tutton E, Griffin X, *et al.* Facilitating trial recruitment: A qualitative study of patient and staff experiences of an orthopaedic trauma trial. *Trials* 2019;**20**:492–492. doi:10.1186/s13063-019-3597-8

4 Kirkby HM. What do potential participants want to know about low risk interventional research? A feasibility study of electronic information provision and a randomised controlled trial of an Interactive Information Sheet (IIS). 2013.

5 Locock L, Smith L. Personal experiences of taking part in clinical trials - A qualitative study. *Patient Educ Couns* 2011;**84**:303–9. doi:10.1016/j.pec.2011.06.002

6 Antoniou EE, Draper H, Reed K, *et al.* An empirical study on the preferred size of the participant information sheet in research. *J Med Ethics* 2011;**37**:557–62. doi:10.1136/jme.2010.041871

7 Usadi RS, Diamond MP, Legro RS, *et al.* Recruitment strategies in two reproductive medicine network infertility trials. *Contemp Clin Trials* 2015;**45**:196–200. doi:10.1016/j.cct.2015.09.010

8 Farquhar C. Endometrial scratching: how much evidence do you need to stop offering this to women having in vitro fertilization? Fertil. Steril. 2019. doi:10.1016/j.fertnstert.2019.04.010

9 Vitagliano A, Andrisani A, Alviggi C, *et al.* Endometrial scratching for infertile women undergoing a first embryo transfer: a systematic review and meta-analysis of published and unpublished data from randomized controlled trials. *Fertil Steril* Published Online First: 2019. doi:10.1016/j.fertnstert.2018.12.008

10 Pye C, Chatters R, Cohen J, *et al.* Induced endometrial trauma (endometrial scratch) in the mid-luteal menstrual cycle phase preceding first cycle IVF/ICSI versus usual IVF/ICSI therapy: Study protocol for a randomised controlled trial. *BMJ Open* Published Online First: 2018. doi:10.1136/bmjopen-2017-020755

11 Redshaw M, Hockley C, Davidson LL. A qualitative study of the experience of treatment for infertility among women who successfully became pregnant. *Hum Reprod* 2007;**22**:295–304. doi:10.1093/humrep/del344

12 Cousineau TM, Domar AD. Psychological impact of infertility. Best Pract. Res. Clin. Obstet. Gynaecol. 2007;**21**:293–308. doi:10.1016/j.bpobgyn.2006.12.003

13 Mourad SM, Hermens RPMG, Cox-Witbraad T, *et al.* Information provision in fertility care: A call for improvement. *Hum Reprod* Published Online First: 2009. doi:10.1093/humrep/dep029

14 Porter M, Bhattacharya S. Helping themselves to get pregnant: A qualitative longitudinal

For peer review only - http://bmjopen.bmj.com/site/about/guidelines.xhtml

study on the information-seeking behaviour of infertile couples. *Hum Reprod* Published Online First: 2008. doi:10.1093/humrep/dem398

15 Meinich Petersen S, Zoffmann V, Kjærgaard J, *et al.* Disappointment and adherence among parents of newborns allocated to the control group: A qualitative study of a randomized clinical trial. *Trials* 2014;**15**:1–11. doi:10.1186/1745-6215-15-126

16 Rooshenas L, Elliott D, Wade J, *et al.* Conveying Equipoise during Recruitment for Clinical Trials: Qualitative Synthesis of Clinicians' Practices across Six Randomised Controlled Trials. *PLoS Med* 2016;**13**. doi:10.1371/journal.pmed.1002147

17 Braun, V., Clark V. Using thematic analysis in psychology. *Qual Res Psychol* 2006;**3**:77–101.

18 Hart A. Randomized controlled trials: The control group dilemma revisited. Complement. Ther. Med. 2001;**9**:40–4. doi:10.1054/ctim.2000.0414

19 Howard L, Thornicroft G. Patient preference randomised controlled trials in mental health research. Br. J. Psychiatry. 2006;**188**:303–4. doi:10.1192/bjp.188.4.303

20 Skingley A, Bungay H, Clift S, *et al.* Experiences of being a control group: lessons from a UK-based randomized controlled trial of group singing as a health promotion initiative for older people. *Health Promot Int* 2014;**29**:751–8. doi:10.1093/heapro/dat026

21 Sheridan R, Martin-Kerry J, Hudson J, *et al.* Why do patients take part in research? An overview of systematic reviews of psychosocial barriers and facilitators. *Trials* 2020;**21**:1–18. doi:10.1186/s13063-020-4197-3

22 Perrotta M, Geampana A. The trouble with IVF and randomised control trials: Professional legitimation narratives on time-lapse imaging and evidence-informed care. *Soc Sci Med* 2020;**258**:113115. doi:10.1016/j.socscimed.2020.113115

23 Paramasivan S, Rogers CA, Welbourn R, *et al.* Enabling recruitment success in bariatric surgical trials: Pilot phase of the By-Band-Sleeve study. *Int J Obes* Published Online First: 2017. doi:10.1038/ijo.2017.153

24 Elliott D, Hamdy FC, Leslie TA, *et al.* Overcoming difficulties with equipoise to enable recruitment to a randomised controlled trial of partial ablation vs radical prostatectomy for unilateral localised prostate cancer. *BJU Int* Published Online First: 2018. doi:10.1111/bju.14432

25 Blencowe NS, Cook JA, Pinkney T, *et al.* Delivering successful randomized controlled trials in surgery: Methods to optimize collaboration and study design. Clin. Trials. 2017. doi:10.1177/1740774516687272

26 Kirkby HM, Calvert M, Draper H, *et al.* What potential research participants want to know about research: A systematic review. BMJ Open. 2012;**2**:e000509. doi:10.1136/bmjopen-2011-000509

27 Jenkins SP, Calvert MJ, Draper H. Potential research participants' use of information during the consent process: A qualitative pilot study of patients enrolled in a clinical trial. *PLoS One* 2020;**15**:e0234388. doi:10.1371/journal.pone.0234388

For peer review only - http://bmjopen.bmj.com/site/about/guidelines.xhtml

COREQ (COnsolidated criteria for REporting Qualitative research) Checklist

A checklist of items that should be included in reports of qualitative research. You must report the page number in your manuscript where you consider each of the items listed in this checklist. If you have not included this information, either revise your manuscript accordingly before submitting or note N/A.

Topic	Item No.	Guide Questions/Description	Reported on Page No.
**Domain 1: Research team and reflexivity**			
Personal characteristics			
Interviewer/facilitator	1	Which author/s conducted the interview or focus group?	4
Credentials	2	What were the researcher's credentials? E.g. PhD, MD	4
Occupation	3	What was their occupation at the time of the study?	4
Gender	4	Was the researcher male or female?	4
Experience and training	5	What experience or training did the researcher have?	4
Relationship with participants			
Relationship established	6	Was a relationship established prior to study commencement?	4
Participant knowledge of the interviewer	7	What did the participants know about the researcher? e.g. personal goals, reasons for doing the research	4
Interviewer characteristics	8	What characteristics were reported about the inter viewer/facilitator? e.g. Bias, assumptions, reasons and interests in the research topic	4
**Domain 2: Study design**			
Theoretical framework			
Methodological orientation and Theory	9	What methodological orientation was stated to underpin the study? e.g. grounded theory, discourse analysis, ethnography, phenomenology, content analysis	5
Participant selection			
Sampling	10	How were participants selected? e.g. purposive, convenience, consecutive, snowball	3,4
Method of approach	11	How were participants approached? e.g. face-to-face, telephone, mail, email	4
Sample size	12	How many participants were in the study?	5, 6
Non-participation	13	How many people refused to participate or dropped out? Reasons?	5, 6
Setting			
Setting of data collection	14	Where was the data collected? e.g. home, clinic, workplace	5
Presence of non-participants	15	Was anyone else present besides the participants and researchers?	5
Description of sample	16	What are the important characteristics of the sample? e.g. demographic data, date	6,7
Data collection			
Interview guide	17	Were questions, prompts, guides provided by the authors? Was it pilot tested?	4
Repeat interviews	18	Were repeat inter views carried out? If yes, how many?	5
Audio/visual recording	19	Did the research use audio or visual recording to collect the data?	5
Field notes	20	Were field notes made during and/or after the inter view or focus group?	5
Duration	21	What was the duration of the inter views or focus group?	5
Data saturation	22	Was data saturation discussed?	5
Transcripts returned	23	Were transcripts returned to participants for comment and/or	5

For peer review only

Topic	Item No.	Guide Questions/Description	Reported on Page No.
		correction?	
**Domain 3: analysis and findings**			
Data analysis			
Number of data coders	24	How many data coders coded the data?	5
Description of the coding tree	25	Did authors provide a description of the coding tree?	5
Derivation of themes	26	Were themes identified in advance or derived from the data?	5
Software	27	What software, if applicable, was used to manage the data?	5
Participant checking	28	Did participants provide feedback on the findings?	5
Reporting			
Quotations presented	29	Were participant quotations presented to illustrate the themes/findings? Was each quotation identified? e.g. participant number	7-10
Data and findings consistent	30	Was there consistency between the data presented and the findings?	7-10
Clarity of major themes	31	Were major themes clearly presented in the findings?	7-10
Clarity of minor themes	32	Is there a description of diverse cases or discussion of minor themes?	7-10

Developed from: Tong A, Sainsbury P, Craig J. Consolidated criteria for reporting qualitative research (COREQ): a 32-item checklist for interviews and focus groups. *International Journal for Quality in Health Care*. 2007. Volume 19, Number 6: pp. 349 – 357

Once you have completed this checklist, please save a copy and upload it as part of your submission. DO NOT include this checklist as part of the main manuscript document. It must be uploaded as a separate file.

For peer review only

BMJ Open

The experiences of trial participants and site staff of participating in and running a large randomised trial within fertility (the Endometrial Scratch Trial) – a qualitative interview study

Journal:	*BMJ Open*
Manuscript ID	bmjopen-2021-051698.R1
Article Type:	Original research
Date Submitted by the Author:	27-Jul-2021
Complete List of Authors:	Chatters, Robin; The University of Sheffield, Clinical Trials Research Unit, School of Health and Related Research<br>White, David; The University of Sheffield, Clinical Trials Research Unit<br>Pye, Clare; Sheffield Teaching Hospitals NHS Foundation Trust, Jessop Wing<br>Petrovic, Ana; Rotherham Doncaster and South Humber Mental Health NHS Foundation Trust<br>Sizer, Anya; Fertility Network UK<br>Kumar, Pavithra; The University of Sheffield, Clinical Trials Research Unit<br>Metwally, Mostafa; Sheffield Teaching Hospitals NHS Foundation Trust
**Primary Subject Heading**:	Reproductive medicine
Secondary Subject Heading:	Research methods
Keywords:	Clinical trials < THERAPEUTICS, Reproductive medicine < GYNAECOLOGY, QUALITATIVE RESEARCH

SCHOLARONE™
Manuscripts

I, the Submitting Author has the right to grant and does grant on behalf of all authors of the Work (as defined in the below author licence), an exclusive licence and/or a non-exclusive licence for contributions from authors who are: i) UK Crown employees; ii) where BMJ has agreed a CC-BY licence shall apply, and/or iii) in accordance with the terms applicable for US Federal Government officers or employees acting as part of their official duties; on a worldwide, perpetual, irrevocable, royalty-free basis to BMJ Publishing Group Ltd ("BMJ") its licensees and where the relevant Journal is co-owned by BMJ to the co-owners of the Journal, to publish the Work in this journal and any other BMJ products and to exploit all rights, as set out in our licence.

The Submitting Author accepts and understands that any supply made under these terms is made by BMJ to the Submitting Author unless you are acting as an employee on behalf of your employer or a postgraduate student of an affiliated institution which is paying any applicable article publishing charge ("APC") for Open Access articles. Where the Submitting Author wishes to make the Work available on an Open Access basis (and intends to pay the relevant APC), the terms of reuse of such Open Access shall be governed by a Creative Commons licence – details of these licences and which Creative Commons licence will apply to this Work are set out in our licence referred to above.

Other than as permitted in any relevant BMJ Author's Self Archiving Policies, I confirm this Work has not been accepted for publication elsewhere, is not being considered for publication elsewhere and does not duplicate material already published. I confirm all authors consent to publication of this Work and authorise the granting of this licence.

For peer review only

Title: The experiences of trial participants and site staff of participating in and running a large randomised trial within fertility (the Endometrial Scratch Trial) – a qualitative interview study

Robin Chatters (corresponding author) – Sheffield Clinical Trials Research Unit, School of Health and Related Research, The University of Sheffield. R.chatters@sheffield.ac.uk

David White - Sheffield Clinical Trials Research Unit, School of Health and Related Research, The University of Sheffield. d.a.white@sheffield.ac.uk

Clare Pye – Assisted Conception Unit, Jessop Wing, Sheffield Teaching Hospitals NHS Foundation Trust. clare.pye1@nhs.net

Ana Petrovic – Rotherham, Doncaster and South Humber NHS Foundation Trust. ana.petrovic@nhs.net

Anya Sizer – Fertility Network UK. anya@fertilitynetworkuk.org

Pavithra Kumar - Sheffield Clinical Trials Research Unit, School of Health and Related Research, The University of Sheffield. p.kumar@sheffield.ac.uk

Mostafa Metwally - Assisted Conception Unit, Jessop Wing, Sheffield Teaching Hospitals NHS Foundation Trust. m.metwally@sheffield.ac.uk

Word count: 4533 words

For peer review only

For peer review only - http://bmjopen.bmj.com/site/about/guidelines.xhtml

ABSTRACT

Objectives: To explore the experiences of endometrial scratch (ES) trial participants and site staff of trial recruitment and participation, in order to improve the experience of participants in future trials.

Design: Qualitative study of a subset of participants in the ES randomised controlled trial (RCT), and a subset of trial site staff.

Setting: A purposeful sample of nine of the 16 UK Fertility Units that participated in the trial.

Participants: A purposeful sample of 27 trial participants and seven site staff.

Results: Participants were largely happy with the recruitment practices, however, some were overwhelmed with the amount of information received. Interviewees had positive preconceptions regarding the possible effect of the ES on the outcome of their IVF cycle, which often originated from their own internet research and seemed to be exacerbated by how site staff described the intervention. Some participants appeared to not understand that receiving the ES could potentially reduce their chances of a successful IVF outcome. Those randomised to the control arm discussed feeling discontent; site staff developed mechanisms of dealing with this.

Conclusions: A lack of equipoise in both study participants and the recruiting site staff led to trial participants having positive preconceptions of the potential impact of the ES on their upcoming IVF cycle. Trial participants may not have understood the potential harms of participating in a randomised trial. The trial information sheet did not clearly state this; further research should assess how such information should be presented to potential participants, to proportionately present the level of risk, but to not unduly discourage participation. The amount of information fertility patients require about a research study should also be investigated, in order to avoid participants feeling overwhelmed by the amount of information they receive prior to starting IVF.

Trial registration: ISRCTN, ISRCTN23800982, post results.

KEYWORDS

Clinical trial; reproductive medicine; qualitative research.

STRENGTH AND LIMITATIONS OF THIS STUDY

- We present data from a purposeful sample of 27 trial participants and seven site staff regarding their experiences of taking part in, and running, the endometrial scratch randomised controlled trial.
- As far as we are aware, this is the first study to investigate the experience of fertility patients of being recruited to, and participating in, a randomised trial.
- We included both participants randomised to the control and intervention arms of the trial, plus those who did and did not have a live birth after their IVF cycle.
- Recall may be an issue, as trial participants were interviewed on average 17.8 months after they were recruited to the trial.
- We only interviewed individuals who participated in the trial, and therefore interviews may represent a biased view of the experiences of being recruited to and participating in the trial.

INTRODUCTION

A wealth of literature exists that qualitatively assesses the experiences of participants being recruited to trials across various disease areas, indicating that participants can find it difficult to understand the potential harms of participating in a study [1,2], and staff experience challenges in expressing equipoise to participants [3]. The participant information sheet (PIS) has been extensively researched; in one study the 'traditional' PIS was found to oversupply information to 79.5% of participants [4].

For peer review only - http://bmjopen.bmj.com/site/about/guidelines.xhtml

Other studies have identified that, if given a choice, participants often opt for a shorter version of the PIS [5,6]. To our knowledge, the experience of patients participating in randomised trials in fertility has not been explored, although recruitment techniques have been described across two trials [7].

The Endometrial Scratch Trial was a randomised controlled trial (RCT) that aimed to assess the clinical and cost effectiveness of undertaking the endometrial scratch prior to first time in vitro fertilisation (IVF). Participants (women recruited between July 2016 and October 2018 from 16 fertility centres across the UK) were randomly allocated to either receive usual IVF treatment, or the endometrial scratch (ES) procedure, followed by usual IVF treatment. The ES involves scratching the lining of the womb (the endometrium) prior to a cycle of IVF and is hypothesised to improve the chances of the embryo implanting by improving the receptivity of the endometrium [8]. A lack of good quality evidence exists to support the delivery of the ES in women undergoing their first cycle of IVF [9]. The full trial protocol is published elsewhere [10].

There are several reasons to investigate the experience of participants and staff within this trial. Undergoing IVF can be a distressing and emotional experience that involves receiving invasive procedures, causing physical and mental stress [11,12]. Patients receive a lot of information prior to their treatment, which has been associated with anxiety [13]. It is therefore important to ensure that participation in research does not create an extra burden for the patient. Furthermore, as women undergoing fertility treatments often seek information from sources other than their fertility team (e.g. the internet, books and magazines), it is pertinent to ascertain how this affected the participant's experience of recruitment to the trial [14]. The study involved potentially difficult decisions for participants, specifically, whether to participate in the trial given the 50% chance of being randomised to a novel intervention with unknown benefits or harms, or the treatment as usual control group. Qualitative evaluations in other trial populations have identified that participants often feel discontent when randomised to the treatment as usual arm [15]. Given that recruiting staff have been found to struggle to convey equipoise in previous studies [3,16], it is interesting to evaluate if these individuals conveyed equipoise, and if this impacted on the participant's experiences of the trial.

The aim of this qualitative study was to understand the experiences of participants being recruited to - and participating in – the endometrial scratch trial, including any issues or barriers they faced. We also aimed to understand trial site staff's experiences, with a general aim to improve the experience for participants in future trials.

METHODS

Sampling, approach and recruitment

Purposive sampling was used to achieve the maximum variation in characteristics across trial participants and staff. Firstly, six trial sites were selected, by seeking distribution of the characteristics described in Table 1 (site sampling attributes). Trial participants who had recently completed the trial and had not withdrawn from trial follow-up were then sampled from these sites, using the attributes presented in Table 1 (participant sampling attributes).

Table 1: Site and participant level sampling attributes

Attribute	Categories
**Site sampling attributes**	
Site type	NHS site/privately owned
Size of centre	number of IVF cycles undertaken per year
Consent rate of centre	the proportion consented relative to those screened

For peer review only - http://bmjopen.bmj.com/site/about/guidelines.xhtml

Participant sampling attributes	
Intervention status	ES received, ES declined, treatment as usual
Age	30 or below, above 30
Duration of infertility	Less than 36 months, 36 months or more
Outcome of first cycle of IVF	Live birth, no live birth

Site staff (principle investigators and research nurses) from the six selected centres were initially sampled for the staff interviews. Due to low staff availability and willingness to participate in the interviews, additional sites were selected as long as the research nurse was still in post and had undertaken recruitment to the trial.

Potential interviewees were initially approached by letter or email. If no contact was received, individuals were telephoned or emailed approximately one week after the initial contact was made. Verbal consent was obtained immediately prior to the interview via telephone, with the conversation regarding consent being audio recorded.

Research team and reflexivity

One woman (AP, research assistant with a PhD) and one man (RC, trial manager with a BSc) conducted the interviews with all participants. Both had prior training and experience of undertaking qualitative interviews. One other female researcher (PK, research assistant with an MSc) assisted with data analysis, and a female patient and public involvement (PPI) representative (AS, with experience of fertility treatments and an employee of a patient charity – Fertility Network UK) reviewed the interview schedule and this manuscript.

RC managed the Endometrial Scratch Trial from the start; AP commenced her involvement more recently prior to the start of this qualitative study. A relationship between site staff and RC was therefore already formed, which was also true for AP but to a lesser extent. Site staff would have been aware of the interviewer's involvement in the design and delivery of the trial and their reasons for doing the research.

The interviewers had no involvement in the clinical care of the trial participants, nor had they had previous contact. Participants would have been aware of the interviewer's reasons for undertaking the research and may or may not have been aware of the interviewers' involvement in the trial in which they participated.

Data collection

A semi-structured interview schedule was used to guide the interviews – with separate schedules being used for trial participants and site staff (see supplementary materials). The schedules were pilot tested in one interview per schedule, and adjustments were made following discussions between AP and RC; the pilot interview is included in the analysis. The participant interviews were undertaken prior to the staff interviews in order to allow themes from the former to be discussed with staff. Prompts were used during interviews and repeat interviews were not carried out.

Interviews were audio recorded and transcribed verbatim. There were no other individuals present at the interviews, except for, in a few instances, the participant's child. The interviewee recorded notes after each interview. Interviews with trial participants ranged from 4.5 to 21.5 minutes, and those with staff were 19.5 to 57.6 minutes.

For peer review only - http://bmjopen.bmj.com/site/about/guidelines.xhtml

The trial participant interview schedule covered the participants' experience of recruitment and participation in the trial. The staff interview schedule included the staff's experience of recruitment and their overall experience of delivering the trial at their site.

All interviews were undertaken by telephone. Data saturation was discussed between RC and AP separately for staff and trial participant interviews.

Data analysis

An inductive thematic analysis was used to analyse the data, which was undertaken within a phenomenological framework. We used Braun and Clark's reflexive thematic analysis framework, which involves six phases of analysis—familiarisation, initial coding, theme construction, reviewing themes, defining themes and producing the report [17]. Two researchers (AP and RC for the trial participant interviews, and PK and RC for the staff interviews) developed the coding structure separately by coding a small number of transcripts (four for the participant interviews and two for the staff interviews). NVivo (v12) was used to undertake all analyses. The researchers then met and resolved any differences in coding in order to develop the final list of codes. All other transcripts were then coded by one researcher, either AP or RC for the trial participant interviews, and RC or PK for the staff interviews. The coding trees were formed of overarching themes that were related to the overall aims of this qualitative study - the experience of receiving/delivering ES, reasons for participants deciding not to receive ES after being randomised to do so, preconceptions of the ES procedure, and experiences of recruitment. However, in this manuscript, the experience of receiving/delivering ES is not reported in order to reflect only those themes that have the potential to improve the experience of participants in future studies. RC undertook all analysis steps after coding, including formation of themes and writing of the report. Interviewees did not feedback on the findings or transcripts. After analysing the transcriptions no new themes emerged, thus data saturation was determined to have been reached.

Patient and public involvement

Input into the design of the study, interview schedules and patient facing materials were sought from the Sheffield Reproductive Health Research Public Advisory Panel and the trial's public involvement representative (AS).

Ethics

This qualitative sub study was approved by Berkshire South Central Research Ethics Committee (REC) (16/SC/0151). All participants gave informed consent before taking part.

RESULTS

Interviews and characteristics of participants

Forty-four trial participants were approached to participate in the study, of which two actively declined to participate and 15 either did not respond or were not contactable. As a result, 27 trial participants took part.

Interviewed participants had an average duration of infertility of three years (range 11 months to 8 years 6 months) and were interviewed, on average, 17.8 months after they were randomised into the trial (range from eight to 33 months). Most participants were in the 33-37 years age range, with ages ranging from 21 to 37 years. Participants were recruited from NHS sites, except for one site (site 6), which was a privately run centre. The demographics of the trial participants that took part in this study are summarised in *Table 2*.

Table 2. Characteristics of interviewed trial participants

ID	Age range at randomisation	Live birth recorded?	Randomisation arm

For peer review only - http://bmjopen.bmj.com/site/about/guidelines.xhtml

Site 1			
Participant 1	33-37	No	ES
Participant 2	33-37	Yes	ES
Participant 3	27-32	No	ES
Participant 4	27-32	No	ES
Participant 5	27-32	No	ES
Site 2			
Participant 1	27-32	Yes	ES
Participant 2	27-32	Yes	ES
Site 3			
Participant 1	33-37	No	ES
Site 4			
Participant 1	33-37	No	ES
Participant 2	33-37	Yes	ES
Participant 3	33-37	Yes	TAU
Participant 4	33-37	No	ES
Site 5			
Participant 1	27-32	Yes	ES
Participant 2	27-32	Yes	TAU
Participant 3	21-26	No	ES
Site 6			
Participant 1	27-32	Yes	TAU
Participant 2	27-32	Yes	ES
Participant 3	33-37	Yes	ES
Participant 4	27-32	No	TAU
Site 7			
Participant 1	33-37	No	ES
Participant 2	33-37	Yes	ES
Participant 3	21-26	No	ES
Participant 4	33-37	Yes	TAU
Site 8			
Participant 1	27-32	No	TAU
Participant 2	33-37	Yes	ES
Participant 3	27-32	No	ES
Participant 4	33-37	No	TAU

ES, endometrial scratch; ID, identification number; N/A, not applicable; TAU, treatment as usual

Seven site staff (one principle investigator (PI) and six research nurses) took part. Fifteen participants were invited (seven PIs and eight research nurses) – one participant actively declined due to lack of time, five participants did not reply contact attempts, and interviews were attempted to be scheduled for two individuals but a mutually agreed time could not be found. The characteristics of the participating site staff can be found in *Table 3*.

Table 3: Characteristics of interviewed site staff

For peer review only - http://bmjopen.bmj.com/site/about/guidelines.xhtml

ID	NHS/Private	Sex
PI-SITE1	NHS	Female
RN-SITE1		Female
RN-SITE3	NHS	Female
RN-SITE4	NHS	Female
RN-SITE5	NHS	Female
RN-SITE6	Private	Female
RN-SITE9	NHS	Female

NHS, National Health Service; PI, Principle Investigator; RN, Research Nurse

Trial participants and site staff discussed four major themes during the interviews –, the experience of recruitment to the trial, the written materials that were provided at recruitment, the lack of equipoise in both trial participants and site staff and the demoralising effect this had on some participants randomised to the control arm, and the reasons for withdrawing from the ES once randomised to receive it. Below, we discuss these themes.

Experience of the general recruitment process

Trial participants were generally happy with the recruitment process.

> *"I think it was all done really well. It was just, it was really well explained and I don't … think, if it was happening again, … I wouldn't change anything about it"* Participant 2 – Site 8

Participating in research whilst undergoing IVF was seen by many as "exciting" and "interesting", and provided an alternative focus for two participants.

> "*I think it's in a way it was actually it was quite a good thing because I think it gave me something else to focus on […] it was quite nice to have something that was being discussed where I felt actually this has got a wider benefit"* Participant 4 – Site 4

Three participants felt that the approach to take part in the trial was too informal, with the participants either having to ask about the trial, or the trial being mentioned at the last minute when there were previous opportunities to raise this.

> *"I don't know if it should be more formal really, because it was just kind of, if they remembered on that day that I would be a possible candidate …, maybe you should get it in a pack or something at the beginning."* Participant 2 – Site 1

Written information provided at recruitment

Four participants could not recall the recruitment materials, due to the time that had elapsed since they were recruited. Of those that could, five participants stated that the materials were clear and informative.

> *"I thought the information sheet was really useful and informative."* Participant 4 – Site 7

There were differences in opinion with regards to the amount of information that trial participants were asked to read during the recruitment process, with some participants stating that the materials were not too onerous to read.

For peer review only - http://bmjopen.bmj.com/site/about/guidelines.xhtml

For peer review only

"*Yeah I think it was maybe like a double sided page it wasn't kind of too much.*" Participant 2 – Site 2

However, others reflected on the amount of information provided more negatively, with two participants discussing the amount of information they received as "overwhelming".

"it can be very overwhelming, having lots of trial information given to you at the same time as all your IVF treatment. It just means that your pack of information to read through, I think I had about twenty different documents or leaflets" Participant 4 – Site 7

One participant suggested that the PIS should contain more information about related studies.

"After reading about other studies in the newspapers like The Guardian, it might have been also interesting to hear a little bit about other studies that were going on… Because, I think when I did read about this study in The Guardian…and it suggested that it wasn't helpful. There was part of me that I think became a little bit angry that maybe I'd put myself through it at the time." Participant 2 – Site 7

Equipoise

Participant's lack of equipoise were exemplified by eighteen of the interviewed participants stating that a major reason to participate in the trial was to increase their chances of a positive outcome from their IVF cycle.

"obviously if they'd have said to us the Scratch trial probably won't have improved our chances of getting pregnant, I probably wouldn't have taken part. So we did it on the basis that we wanted to be on the clinical sample so that we could improve our chances of getting pregnant." Participant 1 – Site 7

Outside of the trial, the privately run site (site 6) offered the ES for a fee, whereas the NHS sites did not offer it at all. Two of the four participants from the private site participated in the trial to receive a 'free' ES. However both of these participants seemed to be well informed regarding the lack of evidence to support the use of ES in this population.

"And they went through all the extras that could be added on to our treatment…they talked about the fact that they were involved in the scratch trial and that it was our first round of IVF. We thought that was probably a good way to do it, rather than to pay for it, if we get lucky and we have it then brilliant and if we don't, you don't yet know how successful it is." Participant 3 – Site 6

There seemed to be a lack of understanding of the potential for the ES to negatively affect the chances of live birth, with five trial participants believing that the ES procedure could not "harm" them.

"There wasn't anything that could potentially harm my chances of success. It wasn't, you know, it wasn't like trying out a new medicine or anything like that" Participant 2 – Site 8

Source of positive preconceptions

Trial participants discussed that a small number of site staff supported their positive preconceptions, sometimes in a subtle way.

"You don't know whether it helps or not but there seems to be this influence from some within the clinic that almost nudge nudge, wink wink, you know,' you're gonna be fine because this really does help." Participant 2 – Site 7

For peer review only - http://bmjopen.bmj.com/site/about/guidelines.xhtml

In other cases, the trial participant reported that they believed site staff thought ES would increase their changes of a live birth

> *"basically they said: "It's completely up to you, it's something that we're giving to you, you can decide." ... obviously they do believe scratches increase chances of success."* Participant 3 – Site 7

However, for some participants, the information provided by site staff ensured they were aware that there was insufficient evidence to suggest that the procedure will improve their chances of success.

> *"The consultant suggested that there wasn't much evidence about whether the scratch was helpful or not when it comes to success of IVF."* Participant 1 – Site 8

Participants also sought information from other sources, most frequently from the internet. For five participants, the internet provided the participant with positive preconceptions of the effect of the ES on their upcoming IVF cycle.

> *"Just the success rate for the people who were trying to do IVF; who were trying to have babies. The success rate after the scratch was high."* Participant 2 – Site 5

However, for two participants, reading information on the internet led them to a position of equipoise.

> "*I wasn't sure, because some studies said it was successful, others said it wasn't, so I was on the fence"* Participant 2 – Site 1

Discontent when randomised to the control arm of the trial

Positive preconceptions of the effect of the ES on the outcome of their IVF cycle led five out of the eight interviewed participants randomised to the control arm feeling some level of discontent with the outcome of randomisation. Some participants discussed feeling slight disappointment.

> *"I think there was a slight bit of disappointment which is ridiculous really because it's randomized. I wasn't sort of like devastated or anything like that... It was, that's what current practice is, to not have the scratch for a first time if under 35 so I was just like well we'll go with that and see what happens. So I didn't dwell on it or anything like that."* Participant 4 – Site 7

For others, randomisation to the control group triggered significant demoralisation.

> *"I was really quite disappointed and quite emotional when I, didn't get on the, Scratch trial itself because I was worried that because I didn't have that, that my chances of getting pregnant were minimized"* Participant 1 – Site 7

Staff awareness of positive preconceptions and demoralisation

Site staff were aware of the trial participant's positive preconceptions.

> "*They would generally say 'oh I've heard about the procedure and it can benefit the chances of you getting pregnant'."* RN-SITE4

In order to attempt to dispel any positive preconceptions during the recruitment process, site staff spent time explaining the lack of an evidence base for ES, and the need for a RCT, to potential trial participants.

> "*we did explain to them comprehensively the evidence, around the Endometrial Scratch and the requirement for a big trial like this to be conducted for us to be able to know the answers*

For peer review only - http://bmjopen.bmj.com/site/about/guidelines.xhtml

and to know whether they should be offered to all patients going through forward for IVF." PI-SITE1

However, site staff reported that participants seemed to struggle to understand the need for a control arm when some studies reported a benefit to having the ES.

"*Sometimes patients found it difficult to accept that if there has been some study that indicate potential benefit, then patients found it difficult to accept that they could be randomised into the control arm."* PI-SITE1

Many site staff were also aware of some participant's disappointment of being randomised to the control arm.

"Some people did express great disappointment and in particularly that way round actually it was nearly always the ones that had wanted it obviously cos it's the intervention." RN-SITE6

Staff described mechanisms they had developed for dealing with this disappointment, summarised in table 4.

Table 4 – methods site staff used to deal with participant's disappointment at being randomised to the control arm of the trial.

**Method of dealing with disappointment**	**Quote**
Explain the importance of the control group	*"So I think its explaining the importance of being in that control group to them. And how we can, you know, we don't know if there was any benefit until we've actually, that's the whole purpose of doing the trial, to answer a question."* RN-SITE1
Describe the lack of evidence to support ES	*"Try and sort of explain that, there was no evidence whether it was a benefit anyway, and that normally if they were going through their treatment, I understand they wouldn't be having a Scratch, so it wasn't as if they missing out on anything."* RN-SITE6
Explain the increased contact with staff that participating in research allows	*"If it's the control you know it's a bit of extra contact with people and I would always say 'look I'm here in the unit, you're not having the Scratch, but if I can help you in any other way, feel free to ring the research mobile. You know to try and always make it I suppose to soften the blow really, and for those who were disappointed."* RN-SITE9

Reasons for withdrawing from the ES

Seven participants were interviewed that, having been randomly allocated to receive the ES, did not receive it. None of the reasons for withdrawing were related to equipoise; five participants declined the ES due to reasons that were out of the control of their fertility centre (illness, holidays, and work commitments). Two participants stated reasons for declining ES that could have been prevented. For one, the site forgot to check the participant's records for a recent smear test result.

"I, we were scheduled to start the IVF and then I got a call from, like the following cycle, I got a call from one of the nurses, not the research nurse one of the other nurses at the facility

For peer review only - http://bmjopen.bmj.com/site/about/guidelines.xhtml

and she said "Oh I've just checked your records and it turns out you're, you're overdue a smear test, we can't proceed with the IVF until you've had a smear test" Participant 1 - Site 5

For the other, the site did not tell the participant to refrain from sexual intercourse in the menstrual cycle in which the ES was being performed. This participant recommended that discussions around abstinence should be more prominent in the recruitment process.

"I think that, that discussion [abstinence from sexual intercourse] needs to be kind of one of the leading things." Participant 1 – Site 1

DISCUSSION AND IMPLICATIONS

Main findings

This qualitative study demonstrates that participants recruited to the Endometrial Scratch Trial were, on the whole, happy with the trial recruitment practices. A small number of participants felt that the invitation to participate in the trial was too informal, and a minority described the amount of written material they received as "overwhelming". There appeared to be a lack of equipoise amongst both sets of interviewees, with regards to the potential effect of the ES on the outcome of the upcoming IVF cycle. Trial participants had positive preconceptions of the ES procedure, which often originated from their own internet research, and in some instances, participants indicated that they believed site staff supported these preconceptions. Some trial participants appeared to not be aware that receiving the ES could potentially reduce their chances of a successful IVF outcome. This, coupled with the positive preconceptions of the ES, led to dissatisfaction amongst some participants randomised to the control arm. Participants from the private centre had a similar experience to those recruited from NHS centres, except that those recruited from the private centre stated a reason to participate in the trial was to receive a free ES, which at the time, was offered at an extra cost by their centre.

Strengths and limitations

A strength of this study is that it included a purposeful sample of women that participated in a large RCT, ensuring variation in trial arm (control or intervention), and live birth status,. We also interviewed staff in order to seek their experiences. A limitation includes that only those that participated in the trial were interviewed, with those participants that withdrew from the trial not being interviewed. This may result in a skewed reflection of the experience of recruitment to the trial. In addition, participants were interviewed, on average, 17.8 months after they agreed to participate in the trial, and furthermore, were interviewed after their IVF cycle had completed. This therefore results in participants reflections on their experiences being clouded by time, and being affected by the outcome of their IVF cycle.

Comparison with existing literature

Our study mirrors the findings of other studies that have described that potential participants have different information needs, and may require different versions of the PIS [5,6]; the discontent felt by participants when randomised to the control group of an RCT [18,19]; participants seeking information from internet sources [14]; and the motivation to receive the intervention has been reported as a major reason participants decide to take part in interventional trials [20,21]. In this study, site staff tried to reposition the participant's positive preconceptions of the ES by explaining the evidence base, which has also been reported in other studies [15,20]. However, it appears that in some cases, participants received cues from site staff that the ES may benefit them, despite having no evidence to support this. Staff have been found to communicate equipoise poorly in other studies, including providing inbalanced descriptions of trial treatments and predicting RCT outcomes [16].

Other studies have also reported that participants overestimate the benefits, and underestimate the risks, of participating in research [1,2]. Our study adds to this evidence base by reporting that, in our study, participants 'arrived' with positive preconceptions, which, in the most part, originated from the internet. This, coupled with subtle signals from recruiting site staff, led to positive preconceptions of the potential effect of the ES procedure on their IVF outcome, and potentially an underestimation of the potential harms of the procedure. This may have been exacerbated by the time-sensitive nature of fertility treatment and the desire for a positive outcome; both clinicians and patients have been

For peer review only - http://bmjopen.bmj.com/site/about/guidelines.xhtml

reported to feel the need to improve the outcomes of treatment immediately following a failed treatment cycle [22].

Implications

Recruiting staff should be aware that participants may base their decision to participate in an RCT on internet research, which risks participants making their own subjective interpretation of the evidence, and may result in overly positive preconceptions of the potential benefits - and a downplaying of the potential harms - of receiving the novel intervention.

Staff should also be aware of subtle cues that may suggest to the participant that the trial intervention may be beneficial to them. The control group should be introduced with similar emphasis to the intervention group, however, discontent once randomised to the control group may still be felt - we present three ways that this was lessened by site staff in our trial (see table 4). Other studies have provided recommendations to guide 'recruitment conversations' in surgical trials – in the opinion of the authors, these guidelines are applicable to studies outside this speciality [23–25].

In order to improve the information provided to participants during the informed consent process, some participants may require more detailed information about the research literature, including the potential harms of the intervention being tested. The authors reflect that the Endometrial Scratch trial PIS did not explicitly state the evidence for or against the ES procedure, nor did it explicitly state the ES may potentially harm the chances of a successful IVF outcome. PPI input was sought into the PIS, but PPI representatives did not comment on the lack of a discussion of the potential harms.

Improvement to trial participant's understanding of the harms of taking part in research, and the role of the control group, may improve the quality of informed consent. This, in turn, may lead to reduced attrition from the trial, especially differential drop out, where a different proportion of participants drop out from one trial arm [26]. Reduced attrition may lead to improved external validity of the trial [27].

Future research

Future research should focus on how the potential harms of taking part in a study can be relayed to participants, in order to deal with overly positive preconceptions. . Details of the potential harms of participating in a study have to be clearly stated in the PIS in clinical trials of an investigational medicinal product (CTIMPs). This issue may have not been given enough consideration in non-CTIMPs; such information could be reviewed by research ethic committees (RECs) prior to providing ethical approval. Checklists to assist PPI representatives in reviewing important patient facing documentation (including a prompt to ensure the potential harms are adequately discussed) could be devised.

Other methods of providing information about the trial to potential participants should be explored, possibly by providing participants with a choice of levels of information, as suggested in previous studies [6,28]. The use of multimedia to inform participants has been assessed within various studies with a trial (SWATs), finding that such mediums did not significantly impact on recruitment to the trial [29,30]. However, importantly the 'quality' of informed consent, and the participant's understanding of the research, was not assessed. Patient acceptable alternatives to the patient information sheet should be investigated, in order to reduce the burden on patients at the start of their fertility treatment.

Footnotes

Acknowledgements: We would like to thank those trial participants and site staff that gave their time and participated in the qualitative interviews.

We would also like to thank the Reproductive Health Research Public Advisory Panel at the Jessop Wing, Sheffield Teaching Hospital NHS Foundation Trust, for their input into the design of this qualitative study.

For peer review only - http://bmjopen.bmj.com/site/about/guidelines.xhtml

Contributors: RC designed the study and led on data collection, analysis and the first draft of the manuscript. DW and CP provided input into the design of the study, analysis and reviewed and approved the manuscript. AS provided input from a lay-person perspective into the design of the study, patient facing materials, results and manuscript. AP inputted into the design of the study, assisted with data collection and analysis, and reviewed and approved the final version of the manuscript. PK assisted with data analysis, and reviewed and approved the final version of the manuscript. MM inputted into the design of the study and reviewed and approved the final version of the manuscript.

Data availability statement: No data are available. The experiences expressed during the interviews refer to sensitive and personally relevant stories; it was agreed that transcripts would not be shared with other researchers in order to preserve the anonymity of study participants.

Competing interests: None declared.

Patient consent for publication: Not required.

Funding: This study is funded by the National Institute for Health Research (NIHR) [Health Technology Assessment Programme (project reference 14/08/45)]. The views expressed are those of the author(s) and not necessarily those of the NIHR or the Department of Health and AQ5 Social Care.

REFERENCES

1 Lidz CW, Appelbaum PS, Grisso T, *et al.* Therapeutic misconception and the appreciation of risks in clinical trials. *Soc Sci Med* 2004;**58**:1689–97. doi:10.1016/S0277-9536(03)00338-1

2 Canvin K, Jacoby A. Duty, desire or indifference? A qualitative study of patient decisions about recruitment to an epilepsy treatment trial. *Trials* 2006;**7**:1–13. doi:10.1186/1745-6215-7-32

3 Phelps EE, Tutton E, Griffin X, *et al.* Facilitating trial recruitment: A qualitative study of patient and staff experiences of an orthopaedic trauma trial. *Trials* 2019;**20**:492–492. doi:10.1186/s13063-019-3597-8

4 Kirkby HM. What do potential participants want to know about low risk interventional research? A feasibility study of electronic information provision and a randomised controlled trial of an Interactive Information Sheet (IIS). 2013.

5 Locock L, Smith L. Personal experiences of taking part in clinical trials - A qualitative study. *Patient Educ Couns* 2011;**84**:303–9. doi:10.1016/j.pec.2011.06.002

6 Antoniou EE, Draper H, Reed K, *et al.* An empirical study on the preferred size of the participant information sheet in research. *J Med Ethics* 2011;**37**:557–62. doi:10.1136/jme.2010.041871

7 Usadi RS, Diamond MP, Legro RS, *et al.* Recruitment strategies in two reproductive medicine network infertility trials. *Contemp Clin Trials* 2015;**45**:196–200. doi:10.1016/j.cct.2015.09.010

8 Farquhar C. Endometrial scratching: how much evidence do you need to stop offering this to women having in vitro fertilization? Fertil. Steril. 2019. doi:10.1016/j.fertnstert.2019.04.010

9 Vitagliano A, Andrisani A, Alviggi C, *et al.* Endometrial scratching for infertile women undergoing a first embryo transfer: a systematic review and meta-analysis of published and unpublished data from randomized controlled trials. *Fertil Steril* Published Online First: 2019. doi:10.1016/j.fertnstert.2018.12.008

10 Pye C, Chatters R, Cohen J, *et al.* Induced endometrial trauma (endometrial scratch) in the

For peer review only - http://bmjopen.bmj.com/site/about/guidelines.xhtml

mid-luteal menstrual cycle phase preceding first cycle IVF/ICSI versus usual IVF/ICSI therapy: Study protocol for a randomised controlled trial. *BMJ Open* Published Online First: 2018. doi:10.1136/bmjopen-2017-020755

11 Redshaw M, Hockley C, Davidson LL. A qualitative study of the experience of treatment for infertility among women who successfully became pregnant. *Hum Reprod* 2007;**22**:295–304. doi:10.1093/humrep/del344

12 Cousineau TM, Domar AD. Psychological impact of infertility. Best Pract. Res. Clin. Obstet. Gynaecol. 2007;**21**:293–308. doi:10.1016/j.bpobgyn.2006.12.003

13 Mourad SM, Hermens RPMG, Cox-Witbraad T, *et al.* Information provision in fertility care: A call for improvement. *Hum Reprod* Published Online First: 2009. doi:10.1093/humrep/dep029

14 Porter M, Bhattacharya S. Helping themselves to get pregnant: A qualitative longitudinal study on the information-seeking behaviour of infertile couples. *Hum Reprod* Published Online First: 2008. doi:10.1093/humrep/dem398

15 Meinich Petersen S, Zoffmann V, Kjærgaard J, *et al.* Disappointment and adherence among parents of newborns allocated to the control group: A qualitative study of a randomized clinical trial. *Trials* 2014;**15**:1–11. doi:10.1186/1745-6215-15-126

16 Rooshenas L, Elliott D, Wade J, *et al.* Conveying Equipoise during Recruitment for Clinical Trials: Qualitative Synthesis of Clinicians' Practices across Six Randomised Controlled Trials. *PLoS Med* 2016;**13**. doi:10.1371/journal.pmed.1002147

17 Braun, V., Clark V. Using thematic analysis in psychology. *Qual Res Psychol* 2006;**3**:77–101.

18 Hart A. Randomized controlled trials: The control group dilemma revisited. Complement. Ther. Med. 2001;**9**:40–4. doi:10.1054/ctim.2000.0414

19 Howard L, Thornicroft G. Patient preference randomised controlled trials in mental health research. Br. J. Psychiatry. 2006;**188**:303–4. doi:10.1192/bjp.188.4.303

20 Skingley A, Bungay H, Clift S, *et al.* Experiences of being a control group: lessons from a UK-based randomized controlled trial of group singing as a health promotion initiative for older people. *Health Promot Int* 2014;**29**:751–8. doi:10.1093/heapro/dat026

21 Sheridan R, Martin-Kerry J, Hudson J, *et al.* Why do patients take part in research? An overview of systematic reviews of psychosocial barriers and facilitators. *Trials* 2020;**21**:1–18. doi:10.1186/s13063-020-4197-3

22 Perrotta M, Geampana A. The trouble with IVF and randomised control trials: Professional legitimation narratives on time-lapse imaging and evidence-informed care. *Soc Sci Med* 2020;**258**:113115. doi:10.1016/j.socscimed.2020.113115

23 Paramasivan S, Rogers CA, Welbourn R, *et al.* Enabling recruitment success in bariatric surgical trials: Pilot phase of the By-Band-Sleeve study. *Int J Obes* Published Online First: 2017. doi:10.1038/ijo.2017.153

24 Elliott D, Hamdy FC, Leslie TA, *et al.* Overcoming difficulties with equipoise to enable recruitment to a randomised controlled trial of partial ablation vs radical prostatectomy for unilateral localised prostate cancer. *BJU Int* Published Online First: 2018. doi:10.1111/bju.14432

25 Blencowe NS, Cook JA, Pinkney T, *et al.* Delivering successful randomized controlled trials in surgery: Methods to optimize collaboration and study design. Clin. Trials. 2017. doi:10.1177/1740774516687272

For peer review only - http://bmjopen.bmj.com/site/about/guidelines.xhtml

26 Bell ML, Kenward MG, Fairclough DL, *et al.* Differential dropout and bias in randomised controlled trials: when it matters and when it may not. *BMJ* 2013;**346**. doi:10.1136/bmj.e8668

27 Molenberghs G, Kenward MG. *Missing Data in Clinical Studies*. Chichester, UK: : John Wiley & Sons, Ltd 2007. doi:10.1002/9780470510445

28 Kirkby HM, Calvert M, Draper H, *et al.* What potential research participants want to know about research: A systematic review. BMJ Open. 2012;**2**:e000509. doi:10.1136/bmjopen-2011-000509

29 Mattock HC, Ryan R, O'Farrelly C, *et al.* Does a video clip enhance recruitment into a parenting trial? Learnings from a study within a trial. *Trials* 2020;**21**:856. doi:10.1186/s13063-020-04779-0

30 Jolly K, Sidhu M, Bower P, *et al.* Improving recruitment to a study of telehealth management for COPD: a cluster randomised controlled 'study within a trial' (SWAT) of a multimedia information resource. *Trials* 2019;**20**:453. doi:10.1186/s13063-019-3496-z

For peer review only

Endometrial Scratch Trial Participant Topic Guide

NOTES: This topic guide is a flexible tool and may be revised as new areas of interest arise during the process of data collection. The wording of questions is for guidance only and can be varied to suit the natural style of the interviewer and the level of understanding of the participant.

ITEMS REQUIRED:

- Audio recorder
- Copies of questionnaires and text messages (or send over to participant via email/post if interview to be undertaken over the phone).
- Consent form (returned via post before interview, or audio recording of consent form undertaken prior to interview)
- PIS (send via email/post prior to interview if telephone interview)
- Look to see if participant received and replied to text messages, paper and/or electronic questionnaires.

RESEARCH AIM

1. What was the reason for participants withdrawing from the intervention?
2. What were recruited trial participant's and research site staff's perceptions of the recruitment process and how did research sites deal with recruiting to two large randomised trials?
3. What were recruited participant's perceptions of the data collection tools used during the trial, specifically, text messages to collect pain and electronic questionnaires?

1) Welcome and context setting:

- Introduce yourself
- Guide the participant through the Participant Information Sheet
- "The interview will last approximately 30 minutes"
- Explain that, "to help us with this study, we would like to make a recording of what we all say today, but nobody will be able to identify you from that recording other than me. Is that okay?"
- Guide the participant through the consent form. Take audio recording of responses (if consent form hasn't been returned already by post)
- "You are free to withdraw at any point and you don't have to answer any of the questions if you don't want to."
- Check they are happy to continue and ask if there are any questions.

2) Proceed to interview:

Research questions (RQ) with associated opening participant questions (PQ) and participant prompts (PP)

Recommended general prompts (Note: list is not exhaustive):

- *Can you please elaborate?*
- *Tell me more.*
- *Could you provide an example please?*

For peer review only - http://bmjopen.bmj.com/site/about/guidelines.xhtml
For peer review only

FOR ALL PARTICIPANTS:

RQ3: What were recruited trial participant's perceptions of the recruitment process and how did research sites deal with recruiting to two large randomised trials?

PQ1: *How were you approached to take part in the Endometrial SCRATCH trial?*

PPa: *How did you feel about the way that you were approached? How was the study introduced to youI? Is there anything that we can do to improve the recruitment process?*

PPb: Do you remember receiving any information about the trial? **If yes,** *how do you feel about the information you had to read about the trial?*

PPc: *If you were to take part again, what information would you need to make the decision to participate? What type of information would be helpful to you?*

PPd: *Why did you choose to take part in the study?*

PPe: *How did you feel about being involved in research while undergoing IVF?*

PPf: *Did you have any preconceptions about the Endometrial Scratch procedure prior to taking part in the trial (i.e. its positive/negative effect)? Do you know where these preconceptions were from?*

PQ2: *Were you approached about any other trials?*

IF YES, PQ3: *How did you find being approached regarding more than one trial?*

PPa: *Did you receive the joint information sheet/letter explaining both e-Freeze and Endometrial Scratch trials? Did you find it useful in informing your decision to participate?*

PPb: *How was the trial/how were the trials introduced to you?*

PPc: *How did you decide which trial to participate in?*

FOR TAU PARTICIPANTS

How did you feel about being randomised to not receive the scratch? (If participant describes feeling of disappointment, sadness, etc.) *Did you speak to staff about your feelings about the outcome of the randomization? How did the staff respond to you? (Did you feel supported? Adequately informed?)*

FOR PARTICIPANTS WHO CHOSE NOT **TO RECEIVE THE INTERVENTION:**

RQ 1: What was the reason for participants withdrawing from the intervention?

PQ4: *Our records show that you chose not to receive the endometrial scratch – can you provide some insight into this please?*

PPa: *Did anything about the way the procedure was introduced to you, or the way it was arranged, influence your decision?*

For peer review only - http://bmjopen.bmj.com/site/about/guidelines.xhtml

PPb: *Can you explain some of the factors that influenced your decision?*

PPc: *How did you feel when you discussed your decision with staff at the IVF unit/clinic?* (note: try and find out if staff could have done any more to facilitate delivery of ES)

FOR PARTICIPANTS WHO RECEIVED THE INTERVENTION:

RQ2: Of those women who did receive the intervention, what were their thoughts of the procedure and the information they received in preparation for the procedure?

PQ6: *How did you find receiving the endometrial scratch?*

PPa: *Would you or would you not have it again? How come?*

PPb: *What did you think of the written/verbal information you received from the fertility unit prior to having the endometrial scratch?*

PPc: *What would you say to other people that are interested in receiving the ES procedure?*

PPd: *How did you feel about your preparedness to receive the procedure? What would have helped with your preparedness, in your opinion?*

PPe: *Should other people receive the procedure?*

FOR ALL PARTICIPANTS:

RQ4: What were recruited participant's perceptions of the data collection tools used during the trial, specifically, text messages to collect pain and electronic questionnaires?

PQ8: *I would now like to ask about the questionnaires and text messages we have sent you to collect information. What did you think of these?*

ONLY THOSE WHO RECEIVED THE INTERVENTION: PPa: *How did you find the pain score text messages that were sent to you?*

PPb: *You received an electronic questionnaire. How did you find completing this? Could it be improved?*

PPc: *Would you rather receive an electronic questionnaire or paper questionnaire?*

PPd: *Which is your preferred way to collect information from you? Electronic or paper?*

For peer review only - http://bmjopen.bmj.com/site/about/guidelines.xhtml

Endometrial Scratch Trial Staff Topic Guide

NOTES: This topic guide is a flexible tool and may be revised as new areas of interest arise during the process of data collection. The wording of questions is for guidance only and can be varied to suit the natural style of the interviewer and the level of understanding of the participant.

ITEMS REQUIRED:

- Audio recorder
- Consent form (send via email/post prior to interview if telephone interview)
- PIS (send via email/post prior to interview if telephone interview)

RESEARCH AIM

What were recruited research site staff's perceptions of the recruitment process and how did research sites deal with recruiting to two large randomised trials?

1) Welcome and context setting:

- Introduce yourself
- Guide the participant through the Participant Information Sheet
- "The interview will last approximately 20 minutes"
- Explain that, "to help us with this study, we would like to make a recording of what we all say today, but nobody will be able to identify you from that recording other than me. Is that okay?"
- Guide the participant through the consent form. If happy to participant, ask to sign (if interview is being undertaken remotely then guide participant through audio consent process).
- "You are free to withdraw at any point and you don't have to answer any of the questions if you don't want to."
- Check they are happy to continue and ask if there are any questions.

2) Proceed to interview:

Question 1: How did you undertake recruitment to the endometrial scratch trial?

Prompts:

How did you decide who to approach?

When did you approach potential participants?

How did you approach potential participants?

Question 2: How did you find explaining randomisation, and the outcome of randomisation, to trial participants?

Prompts:

How did you explain randomisation?

Did you encounter any challenges in explaining randomisation?

How did you find participants comprehension of randomisation?

For peer review only - http://bmjopen.bmj.com/site/about/guidelines.xhtml

How did you experience informing participants they were randomised to the control arm?

Did of any your participants have a negative response/reaction to being randomised to the control arm? How did you deal with this?

Question 3: How did you find the recruitment materials? Did you need to supplement these with any additional information?

Prompts:

How did you find the trial PIS/consent form/video in informing the participant about the trial and then ES?

Did you provide the participant with any additional information regarding ES not contained within the PIS/consent form/video? Did they signpost the participants to any other resources?

How did you deal with participants who struggled to understand key aspects of the trial (e.g. randomisation or intervention)

Did you use pictures/diagrams?

Did you provide participants with any additional details in order to aid their understanding/decision to participate in the trial?

Question 4: What were the challenges you encountered over the course of the trial?

Prompts:

Do you think your IVF unit was suitably set up to run this trial? (knowledge and skill level) Did you have capacity to run the current trial? (resources) Would you have capacity to integrate further trials at the same time?

Question 5: Did recruitment practices or your opinion of the intervention change over the course of the trial?

Prompts:

Did you or did you not have any preference for the endometrial scratch for improving success rates? What are your current opinions on the ES? How has the changed since the start of the trial?

How did newly published research during the course of the trial affect your recruitment practices and your opinion of the ES?

Did participants enquire about the new research? Both those newly screened and already in the trial?

Question 6: How did you discuss the endometrial scratch procedure with participants?

Prompts:

Did you encounter any issues with participants concerned that participation in the trial would cause delays to IVF? How did you deal with this?

How did you discuss recovery from the procedure?

Did participants enquire regarding opportunities to receive ES outside of the trial? How did you deal with this?

Did you inform participant whether or not they could receive ES outside of the trial?

For peer review only - http://bmjopen.bmj.com/site/about/guidelines.xhtml

Question 7: Prior to taking part in the trial, did participants have any expectations or preconceptions of ES?

Prompts:

Did participants have any knowledge of ES at the time of entering the study?

How did you manage those expectations?

Did you notice that the participant had any positive or negative preconceptions?

Question 8: How did you find your site managed the scheduling of the ES procedure?

Question 9: Did any of your participants decline the ES? How did you deal with this?

Prompts:

If participants were unable to attend an initial ES procedure, how did you respond?

Question 10: How did you manage participants pain during and post ES procedure?

Prompts:

What support did you provide particpants post procedure?

Did pariticipants believe that any post procedure phenomena were linked to ES?

Was your centre recruiting to more than one trial at once?

IF YES: Question 11: How did you find recruiting to multiple studies? How did you chose which participant to approach each trial?

Prompts:

Did you use the joint information sheets and letters?

Did you have any systems set up in order to recruit to both trials?

What do you think that patients thought about being approached to two large randomised trials?

Question 12: How did you manage negative pregnancy outcomes? Did participants present with any concerns that their randomisation outcome affected the success of their IVF?

Question 13: How did you find using the database and communicating with the central trial management team?

Prompts:

Weekly emails – too much information? Database – any ways of making more user friendly? Would prefer more email/phone/in person contact?

For peer review only

For peer review only - http://bmjopen.bmj.com/site/about/guidelines.xhtml

COREQ (COnsolidated criteria for REporting Qualitative research) Checklist

A checklist of items that should be included in reports of qualitative research. You must report the page number in your manuscript where you consider each of the items listed in this checklist. If you have not included this information, either revise your manuscript accordingly before submitting or note N/A.

Topic	Item No.	Guide Questions/Description	Reported on Page No.
**Domain 1: Research team and reflexivity**			
Personal characteristics			
Interviewer/facilitator	1	Which author/s conducted the interview or focus group?	4
Credentials	2	What were the researcher's credentials? E.g. PhD, MD	4
Occupation	3	What was their occupation at the time of the study?	4
Gender	4	Was the researcher male or female?	4
Experience and training	5	What experience or training did the researcher have?	4
Relationship with participants			
Relationship established	6	Was a relationship established prior to study commencement?	4
Participant knowledge of the interviewer	7	What did the participants know about the researcher? e.g. personal goals, reasons for doing the research	4
Interviewer characteristics	8	What characteristics were reported about the inter viewer/facilitator? e.g. Bias, assumptions, reasons and interests in the research topic	4
**Domain 2: Study design**			
Theoretical framework			
Methodological orientation and Theory	9	What methodological orientation was stated to underpin the study? e.g. grounded theory, discourse analysis, ethnography, phenomenology, content analysis	5
Participant selection			
Sampling	10	How were participants selected? e.g. purposive, convenience, consecutive, snowball	3,4
Method of approach	11	How were participants approached? e.g. face-to-face, telephone, mail, email	4
Sample size	12	How many participants were in the study?	5, 6
Non-participation	13	How many people refused to participate or dropped out? Reasons?	5, 6
Setting			
Setting of data collection	14	Where was the data collected? e.g. home, clinic, workplace	5
Presence of non-participants	15	Was anyone else present besides the participants and researchers?	5
Description of sample	16	What are the important characteristics of the sample? e.g. demographic data, date	6,7
Data collection			
Interview guide	17	Were questions, prompts, guides provided by the authors? Was it pilot tested?	4
Repeat interviews	18	Were repeat inter views carried out? If yes, how many?	5
Audio/visual recording	19	Did the research use audio or visual recording to collect the data?	5
Field notes	20	Were field notes made during and/or after the inter view or focus group?	5
Duration	21	What was the duration of the inter views or focus group?	5
Data saturation	22	Was data saturation discussed?	5
Transcripts returned	23	Were transcripts returned to participants for comment and/or	5

For peer review only

Topic	Item No.	Guide Questions/Description	Reported on Page No.
		correction?	
**Domain 3: analysis and findings**			
Data analysis			
Number of data coders	24	How many data coders coded the data?	5
Description of the coding tree	25	Did authors provide a description of the coding tree?	5
Derivation of themes	26	Were themes identified in advance or derived from the data?	5
Software	27	What software, if applicable, was used to manage the data?	5
Participant checking	28	Did participants provide feedback on the findings?	5
Reporting			
Quotations presented	29	Were participant quotations presented to illustrate the themes/findings? Was each quotation identified? e.g. participant number	7-11
Data and findings consistent	30	Was there consistency between the data presented and the findings?	7-11
Clarity of major themes	31	Were major themes clearly presented in the findings?	7-11
Clarity of minor themes	32	Is there a description of diverse cases or discussion of minor themes?	7-11

Developed from: Tong A, Sainsbury P, Craig J. Consolidated criteria for reporting qualitative research (COREQ): a 32-item checklist for interviews and focus groups. *International Journal for Quality in Health Care*. 2007. Volume 19, Number 6: pp. 349 – 357

Once you have completed this checklist, please save a copy and upload it as part of your submission. DO NOT include this checklist as part of the main manuscript document. It must be uploaded as a separate file.

For peer review only
